# Temporal requirements for ISL1 in sympathetic neuron proliferation, differentiation, and diversification

**DOI:** 10.1038/s41419-018-0283-9

**Published:** 2018-02-14

**Authors:** Qingquan Zhang, Ru Huang, Youqiong Ye, Xiaoxia Guo, Jun Lu, Fugui Zhu, Xiaohui Gong, Qitong Zhang, Jie Yan, Lina Luo, Shaowei Zhuang, Yihan Chen, Xiaodong Zhao, Sylvia M. Evans, Cizhong Jiang, Xingqun Liang, Yunfu Sun

**Affiliations:** 1 Key Laboratory of Arrhythmia, Ministry of Education, East Hospital, Tongji University School of Medicine, Shanghai, China; 2 School of Life Sciences and Technology, Tongji University, Shanghai, China; 3 Key Laboratory of Systems Biomedicine, Ministry of Education, Shanghai Centre for Systems Biomedicine, Shanghai Jiao Tong University, Shanghai, China; 4 Seventh People’s Hospital of Shanghai University of TCM, Shanghai, China; 5 Department of Medicine, Department of Pharmacology, Skaggs School of Pharmacy, University of California San Diego, California, USA

## Abstract

Malformations of the sympathetic nervous system have been associated with cardiovascular instability, gastrointestinal dysfunction, and neuroblastoma. A better understanding of the factors regulating sympathetic nervous system development is critical to the development of potential therapies. Here, we have uncovered a temporal requirement for the LIM homeodomain transcription factor ISL1 during sympathetic nervous system development by the analysis of two mutant mouse lines: an *Isl1* hypomorphic line and mice with *Isl1* ablated in neural crest lineages. During early development, ISL1 is required for sympathetic neuronal fate determination, differentiation, and repression of glial differentiation, although it is dispensable for initial noradrenergic differentiation. ISL1 also plays an essential role in sympathetic neuron proliferation by controlling cell cycle gene expression. During later development, ISL1 is required for axon growth and sympathetic neuron diversification by maintaining noradrenergic differentiation, but repressing cholinergic differentiation. RNA-seq analyses of sympathetic ganglia from *Isl1* mutant and control embryos, together with ISL1 ChIP-seq analysis on sympathetic ganglia, demonstrated that ISL1 regulates directly or indirectly several distinct signaling pathways that orchestrate sympathetic neurogenesis. A number of genes implicated in neuroblastoma pathogenesis are direct downstream targets of ISL1. Our study revealed a temporal requirement for ISL1 in multiple aspects of sympathetic neuron development, and suggested *Isl1* as a candidate gene for neuroblastoma.

## Introduction

Neurons of the sympathetic nervous system (SNS) innervate visceral organs, glands, and blood vessels, and play important roles in maintaining homeostasis. Aberrant development of the SNS causes neurocristopathies associated with cardiovascular instability, gastrointestinal dysfunction, as well as neuroblastoma that arises from developing sympathetic neurons^1^. The SNS is derived from trunk neural crest (NC) cells. As NC cells commit to the sympathetic lineage, they downregulate the expression of genes characteristic of NC cells such as *Sox10*, and express several sympathetic neuron-specific genes such as *Phox2b* and markers of catecholamine biosynthesis (TH, DBH). The newly generated sympathetic neurons transiently exit the cell cycle, and then reenter the cell cycle and are proliferating. Thereafter, an increasing number of sympathetic neurons exit the cell cycle, which peaks at embryonic day 14.5 (E14.5), and undergo post-mitotic differentiation^2–5^.

Bone morphogenetic protein (BMP) and Notch signals are required for induction and coordination of early sympathetic differentiation and progenitor maintenance. Enhanced Notch signaling results in increased Sox10+ progenitors at the expense of differentiated neurons in sympathetic ganglia^6–9^. BMPs initiate sympathetic neurogenesis by inducing sequential expression of a number of transcription factors^2–4^, including Phox2a/2b, Mash1, Hand2, Gata2/3, and Insm1. In Phox2b knockout mice, NC progenitors fail to initiate sympathetic differentiation and express Hand2, Gata3, TH, and DBH^10^. Gata2/3, Insm1, and Hand2 are BMP-induced transcription factors essential for sympathetic neuron proliferation and differentiation downstream of Mash1 and Phox2b. Together, these factors constitute an early transcriptional network essential for sympathetic neuron development^2–4^.

Segregation of initially bimodal sympathetic neurons into distinct noradrenergic and cholinergic neurons occurs relatively later during development, and is controlled by intrinsic as well as target derived signals^11,12^. Recent studies have uncovered a cross-repressive and maintenance mechanism for sympathetic neuron diversification, which involves antagonistic actions between pro-cholinergic factors Ntrk3 (TrkC)/Ret/Tlx3 and pro-noradrenergic factors Hmx1/Ntrk1 (TrkA)^13,14^. TrkC and Ret repress Hmx1 expression and induce cholinergic differentiation. Conversely, Hmx1 represses Tlx3 and Ret, induces TrkC, and maintains noradrenergic phenotypes.

ISL1 is an LIM-homeodomain transcription factor expressed in all peripheral neurons^15^. ISL1 plays an essential role in sensory neuron differentiation by activating genes critical for sensory neuron phenotype, and repressing genes expressed earlier during sensory neuron development^15^. ISL1 is required for early sympathetic neuron development^16^; however, genes and signaling pathways regulated by ISL1 and direct targets of ISL1 in sympathetic neurons remain unexplored. In this study, we uncovered a temporal requirement for ISL1 in various aspects of SNS development. ISL1 regulates directly or indirectly a number of genes essential for sympathetic neuron proliferation and differentiation, many of which have been implicated in neuroblastoma pathogenesis, suggesting *Isl1* as a candidate gene for neuroblastoma.

## Results

### ISL1 is required for proliferation and survival of sympathetic neurons at early developmental stages

**Fig. 1:**
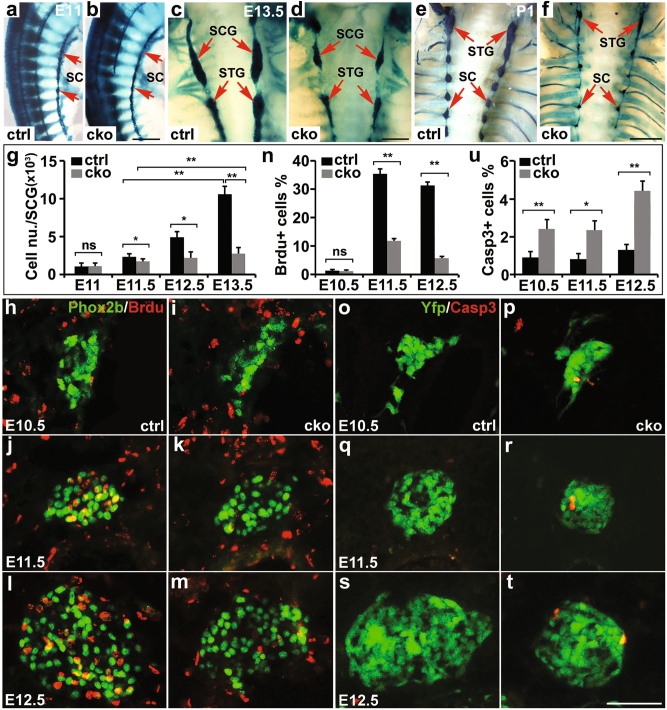
Neural crest specific ablation of *Isl1* leads to loss of sympathetic neurons. **a**, **b** Wholemount X-gal staining revealed that initial formation of primary sympathetic ganglia appeared to be normal in *Wnt1-Cre;Isl1*^*f/f*^ embryos (CKO) compared with *Wnt1-Cre;Isl1*^*+/+*^ (ctrl) littermates at E11. Scale bar, 500 µm. **c**–**f** From E11.5 onward, the size of the superior cervical ganglia (SCG), stellate ganglia (STG), and sympathetic chain (SC) of *Isl1* CKO embryos became progressively reduced. Scale bar, 1 mm (**c**, **d**), 2 mm (**e**, **f**). **g** Quantitative analysis revealed a significant reduction in the total number of sympathetic neurons (X-gal+) per ganglion or sympathetic chain (E11) in *Isl1* CKO embryos compared with control littermates at all developmental stages examined except E11. Error bars represent the s.d., *n* = 4, E11 (*p* = 0.54), E11.5 (*p* = 0.0183), E12.5 (*p* = 0.0106), E13.5 (*p* = 2e−05), two-tailed *t*-test. **h**–**n** BrdU labeling revealed a significantly reduced proliferation of *Isl1* CKO sympathetic neurons (Phox2b+) at E11.5 and E12.5. At E10.5, no BrdU immunoreactivity was detected in both control and CKO mutant embryos. Error bars represent the s.d., *n* = 4, E10.5 (*p* = 0.594), E11.5 (*p* = 6.6e−06), E12.5 (*p* = 4.9e−08), two-tailed *t*-test. Scale bar, 50 µm. **o**–**u** Caspase-3 immunostaining showed significantly increased cell death in the SCG of *Isl1* CKO embryos (on Rosa-YFP background) at E10.5, E11.5, and E12.5. Error bars represent the s.d., *n* = 4, E10.5 (*p* = 0.0016), E11.5 (*p* = 0.0103), E12.5 (*p* = 0.0002), two-tailed *t*-test. Scale bar, 50 µm. Asterisks (*) indicate statistically significant difference between the two groups indicated in brackets, ns = not significant, * *p* < 0.05, or ** *p* < 0.01

We performed wholemount X-gal staining of *Wnt1-Cre; Isl1*^f/f^;*Rosa26-LacZ* mutant (CKO) and *Wnt1-Cre;Isl1*^*+/+*^*;Rosa26-LacZ* control (ctrl) embryos, and found that primary sympathetic column/chain of *Isl1* CKO and ctrl embryos appeared comparable up until E11 (Fig. 1a, b). Thereafter, however, the size of *Isl1* CKO sympathetic ganglia became progressively reduced (Fig. 1c–f). At E10.5–11, the number of sympathetic neurons was comparable between *Isl1* CKO and control embryos (Fig. 1g). However, from E11.5 to E14.5, the number of *Isl1* CKO sympathetic neurons was significantly decreased compared to controls (Fig. 1g).

Sympathetic neurons pause cell division at E10.5, and then reenter the cell cycle from E11.5 onward, until their terminal differentiation during later development^5^. Consistent with this, at E10.5, we observed few, if any, proliferating sympathetic neurons (BrdU+/Phox2b+) in either *Isl1* CKO or control sympathetic ganglia (Fig. 1h, i, and n). At E11.5–12.5, however, we observed a significant reduction in the number of BrdU+ cells in *Isl1* mutant sympathetic ganglia compared to controls at E11.5 (~11% vs. 35%) and E12.5 (~7% vs. 30%) (Fig. 1j–n). In addition, cleaved Caspase-3 immunostaining revealed a significant increase in cell death in *Isl1* CKO sympathetic ganglia at E10.5–E12.5 (Fig. 1o–u).

### RNA-seq analysis reveals downregulation of a large number of genes essential for cell cycle progression at early developmental stages

**Fig. 2:**
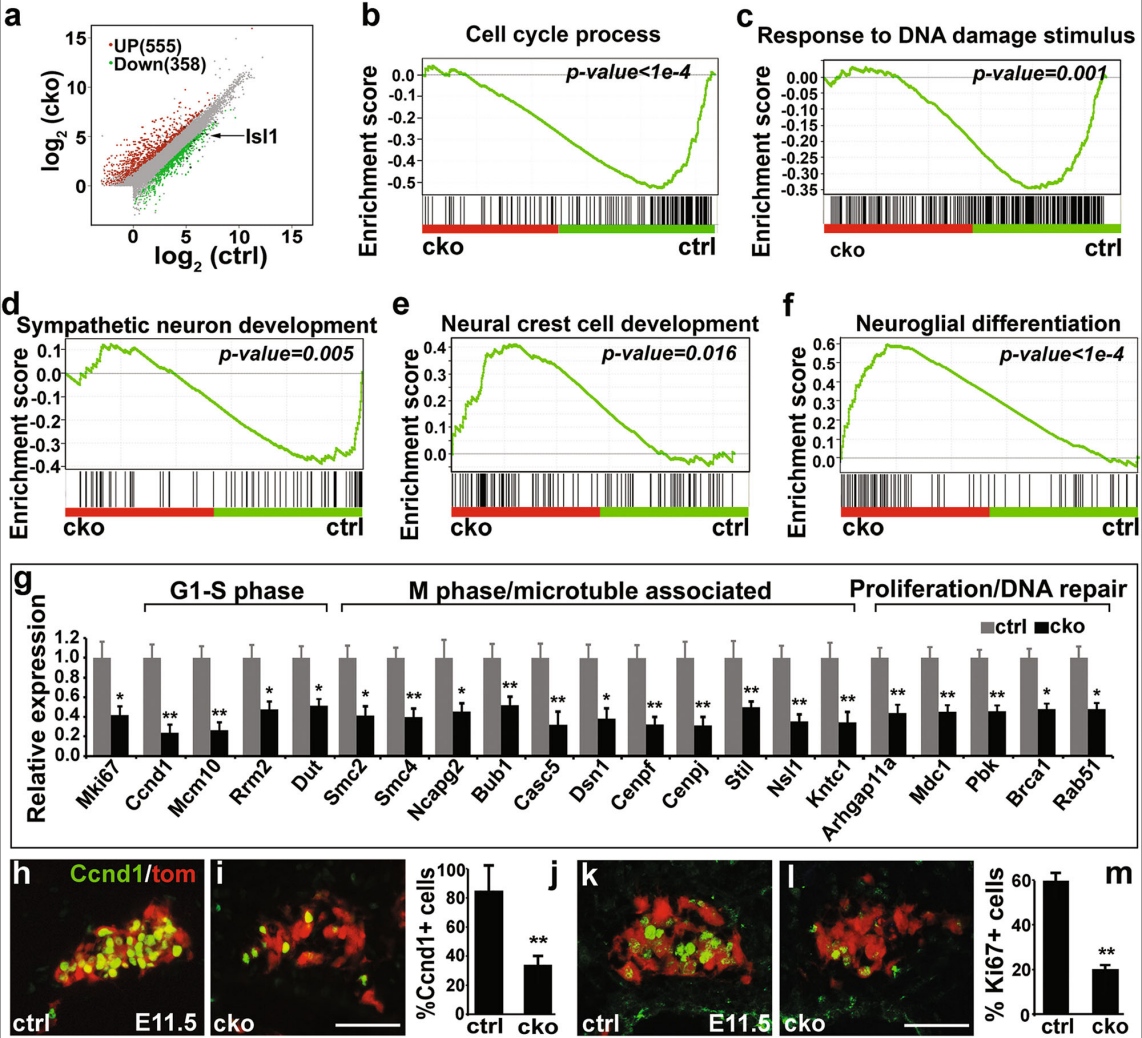
RNA-seq analyses reveal dysregulation of genes essential for sympathetic neuron proliferation in *Isl1* CKO embryos. **a** Scatterplot illustrating relative gene expression of polyA-selected RNA transcripts from RNA-seq comparison of wildtype control and *Isl1* CKO mutant sympathetic neurons, and showing 555 genes upregulated (red) and 358 genes downregulated (green) (| fold change mutant vs. ctrl | ≥ 1.5, *p* < 0.05) in *Isl1* mutant sympathetic neurons. **b**–**f** GSEA showing downregulated gene sets representing cell cycle, response to DNA damage stimulus, and sympathetic neuron development, but upregulated gene sets representing neural crest cell development and neuroglial differentiation. **g** qPCR validation of selected genes involved in the cell cycle and proliferation. Results are shown as relative expression CKO vs. ctrl. Error bars represent the s.d. *n* = 3, *p* < 0.05*, or *p* < 0.01**, two-tailed *t*-test. **h**–**m** Immunostaining and quantitative analysis showing a marked reduction in expression of Mki67 and Ccnd1 in *Isl1* CKO sympathetic ganglia marked by *Rosa26-Tomato* compared with controls at E11.5. Error bars represent the s.d., *n* = 3, *p* = 0.0008 (**j**), *p* = 0.0001 (**m**), two-tailed *t*-test. Scale bar, 50 µm. * *p* < 0.05, or ** *p* < 0.01

To better understand the genetic program regulated by ISL1, we performed RNA-seq analysis on sympathetic ganglia of *Isl1* CKO and control embryos at E11.5–12, a stage when minimal cell loss occurred in *Isl1* CKO sympathetic ganglia. We identified 358 downregulated and 555 upregulated transcripts in *Isl1* CKO mutants (|fold change *Isl1* CKO vs. ctrl | ≥ 1.5, *p* ≤ 0.05) (Fig. 2a and Supplementary Table 1). Gene Set Enrichment Analysis (GSEA) revealed significant downregulation of gene sets representing the cell cycle, response to DNA damage stimulus, and sympathetic neuron development, but upregulation of gene sets representing NC development and glial differentiation (Fig. 2b–f). Selected cell cycle genes were verified by qPCR on FACS sorted sympathetic neurons from E11.5 *Isl1* CKO and control embryos (*Rosa26-tdTomato*+). These included, e.g., *Mki67* (all phases), *Ccnd1* (G1-S phase), *Smc2/4*, *Bub1* (M phase), *Arhgap11a*, *Pbk* (proliferative signaling) (Fig. 2g). Reduced expression of *Ccnd1* and *Mki67* was further confirmed by immunostaining (Fig. 2h–m).

### ISL1 is required for effective downregulation of NC and glial progenitor factors during initial sympathetic neuronal differentiation

**Fig. 3:**
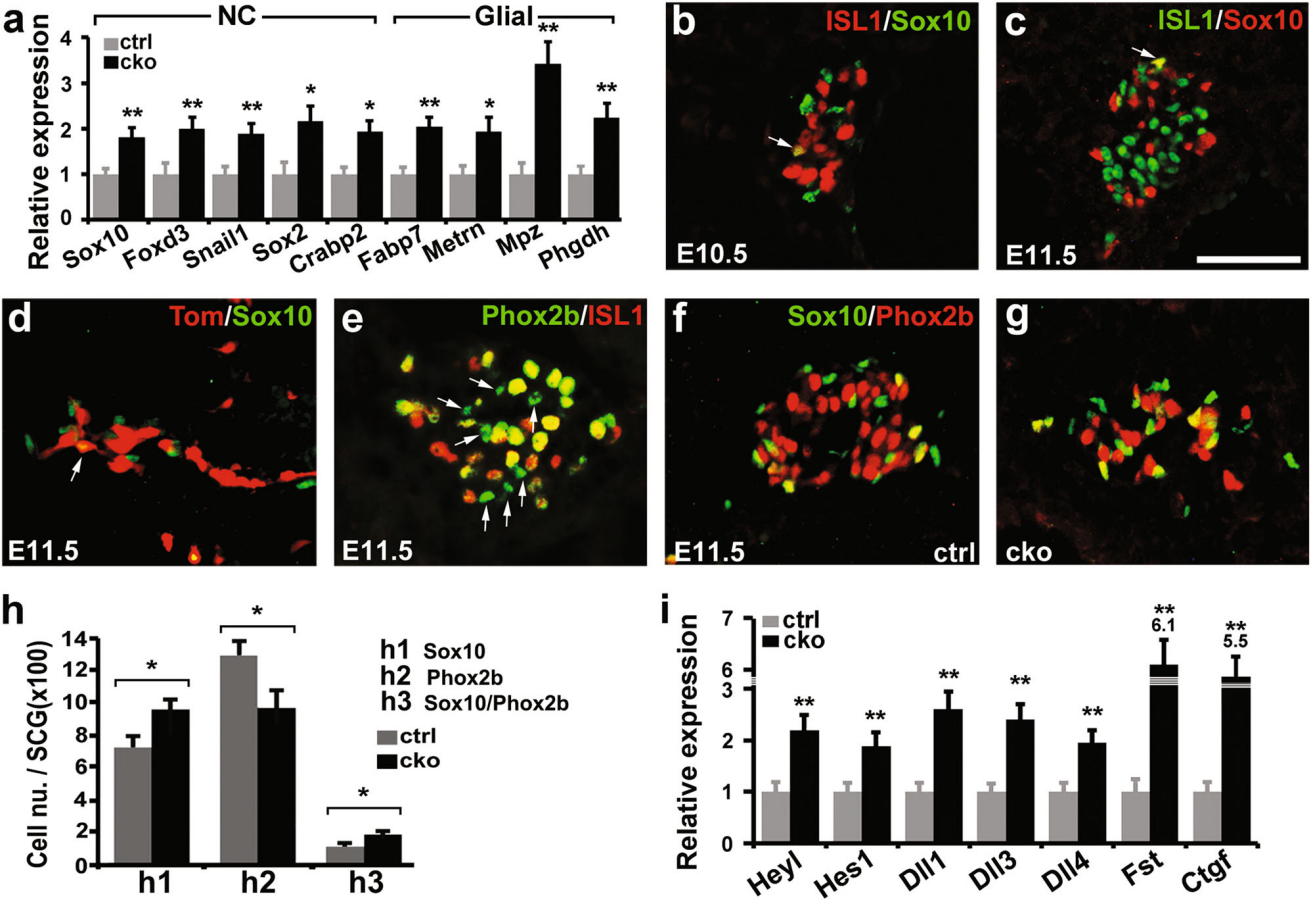
Impaired sympathetic neurogenesis but increased neural crest and glial progenitors in *Isl1* CKO embryos. **a** qPCR verification of upregulated genes characteristic of NC and glial progenitors at E11.5–12. Error bars represent the s.d., *n* = 3, two-tailed *t*-test. **b**–**e** Co-immunostaining of ISL1/Sox10, Tom/Sox10, and Phox2b/ISL1 at E10.5 and E11.5. Scale bar, 50 µm. **f**–**h** Co-immunostaining of Sox10 and Phox2b in *Isl1* CKO and control sympathetic ganglia showing a significant decrease in the number of Phox2b+ neurons (**h2**), but a significant increase in the number of Sox10+ (**h1**) and Phox2b+/Sox10+ (**h3**) progenitors. Error bars represent the s.d., *n* = 4, *p* = 0.0135 (**h1**), *p* = 0.0109 (**h2**), *p* = 0.0134 (**h3**), two-tailed *t*-test. Scale bar, 50 µm. **i** qPCR verification of upregulation of a number of genes of Notch signaling pathway and BMP antagonists at E11.5–12. Error bars represent the s.d., *n* = 3, two-tailed *t*-test. * *p* < 0.05, or ** *p* < 0.01

Our RNA-seq and qPCR analyses revealed significant upregulation of genes associated with NC (*Sox10*, *Foxd3*, *Snai1*, *Sox2*, and *Crabp2*) and glial (*Fabp7*, *Metrn*, *Mpz*, and *Phgdh*) progenitors (Fig. 3a and Supplementary Table 1), suggesting a failure in their developmental downregulation in sympathetic neuroblasts, and an increase in gliogenesis. To investigate the role of ISL1 in early sympathetic neurogenesis, we further analyzed co-expression of Sox10 (NC and glial progenitors, and sympathetic neuronal progenitors), Phox2b (sympathetic progenitors and neurons), and ISL1 during early sympathetic development. At E10.5 and 11.5, a proportion of sympathetic progenitors co-expressed Phox2b and Sox10; however, few, if any, ISL1-expressing or *Isl1-Cre* lineage-labeled sympathetic neurons co-expressed Sox10 (0–1 per section) (Fig. 3b–d, arrow). At E11.5, all ISL1-expressing sympathetic neurons co-expressed Phox2b; however, a subset of Phox2b+ cells did not express ISL1 and were presumably progenitors for NC, sympathetic neurons, or glia (Fig. 3e, arrow). In *Isl1* CKO sympathetic ganglia, the number of Phox2b+ sympathetic neurons was decreased; however, the number of Sox10+ cells, and Sox10+/Phox2b+ progenitors was significantly increased (Fig. 3f–h).

BMP and Notch signals are required for coordinating sympathetic differentiation and progenitor maintenance^6–9^. We found that ablation of *Isl1* during early sympathetic development resulted in reduced BMP signaling (Bmpr1b), but upregulated BMP antagonist signaling (*Ctgf* and *Fst*), and a significant increase in expression of Notch ligands (*Dll1*, *Dll3*) and downstream target genes (*Hes1*, *Heyl*, and *Sox2*)^17^ (Fig. 3i and Supplementary Table 1). Together, these data suggest that ISL1 plays an important role in initial sympathetic neurogenesis, likely by modulating BMP and Notch signaling pathways.

### Role of *Isl1* in the gene regulatory network essential for sympathetic neuron differentiation at early developmental stages

**Fig. 4:**
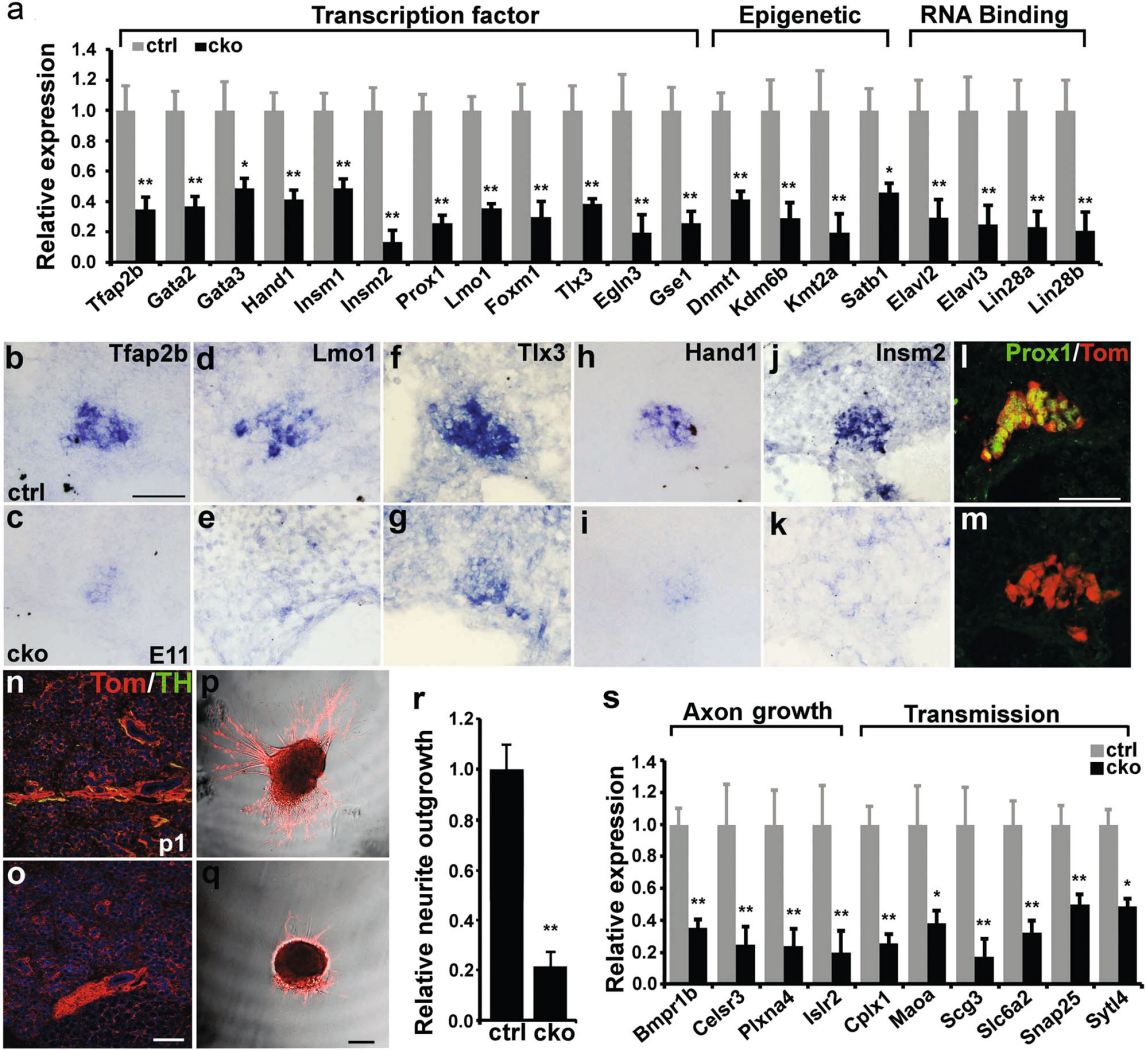
Impaired differentiation and axon growth of sympathetic neurons in *Isl1* CKO embryos. **a** qPCR analysis showing downregulation of genes involved in transcription, epigenetic regulation, and RNA binding on sympathetic ganglia of *Isl1* CKO and control embryos at E11.5–E12. Error bars represent the s.d., *n* = 3, two-tailed *t*-test. **b**–**m** Selected genes downregulated in *Isl1* CKO sympathetic ganglia were verified by in situ hybridization (**b**–**k**) or immunostaining (**l**, **m**) in SCG at E11. Scale bar, 50 µm. **n**, **o** TH+ immunostaining showing the absence of sympathetic innervation of the salivary gland in *Isl1* CKO mice compared with controls. Scale bar, 200 µm. **p**–**r** Sympathetic ganglion explants culture showing significantly reduced axon growth in *Isl1* CKO mutants compared with controls. Error bars represent the s.d., *n* = 3, *p* = 0.0005, two-tailed *t*-test. Scale bar, 100 µm. **s** qPCR confirming downregulation of a number of genes involved in axon growth and neurotransmission. Error bars represent the s.d., *n* = 3, two-tailed *t*-test. * *p* < 0.05, or ** *p* < 0.01

Our RNA-seq data revealed significant downregulation of genes involved in neuronal development. We further examined significantly downregulated genes for those that might contribute to the observed phenotypes of *Isl1* CKO SNS, confirming downregulation in their expression by qPCR on E11.5 sympathetic neurons (Fig. 4a). These included genes involved in transcriptional regulation (e.g., *Gata2/3, Insm2, Tlx3*), chromatin modification (e.g., *Dnmt1, Kdm6b*), and RNA binding (*Elavl2/3, Lin28a/b*), many of which are known to play critical roles in sympathetic neuron development^2–4^ (Fig. 4a). Downregulation of selected genes was further confirmed by in situ hybridization or by immunostaining (Fig. 4b–m).

To understand the role of ISL1 in sympathetic axon development and neurotransmission, we examined sympathetic innervation by TH immunostaining. We observed a marked reduction or nearly absence of sympathetic innervations in the heart at E14.5 and E17.5 (Supplementary Figure 1a-d) and the salivary gland at P1 (Fig. 4o) in *Isl1* CKO mice compared to controls. However, defects in sympathetic innervation may be secondary to neuronal loss in *Isl1* CKO sympathetic ganglia. Therefore, we cultured sympathetic ganglia from E12 *Isl1* CKO and control mice, and axon growth was assessed after 24–48 h. We observed a significant 5-fold reduction in neurite outgrowth in *Isl1* CKO mutants compared to controls (Fig. 4q, r). Furthermore, RNA-seq analysis revealed significantly altered expression of genes involved in neuronal projection morphogenesis and function. qPCR confirmed downregulation of genes involved in axon growth (e.g., *Bmpr1b, Plxna4*), neurotransmission (e.g., *Cplx1, Scg3, Snap25*), and altered expression of genes of neurotransmitters or related enzymes (Fig. 4s; Supplementary Figure 1e and f).

### Reduced *Isl1* expression leads to impaired sympathetic innervation, and delayed cell cycle withdrawal during later development

**Fig. 5:**
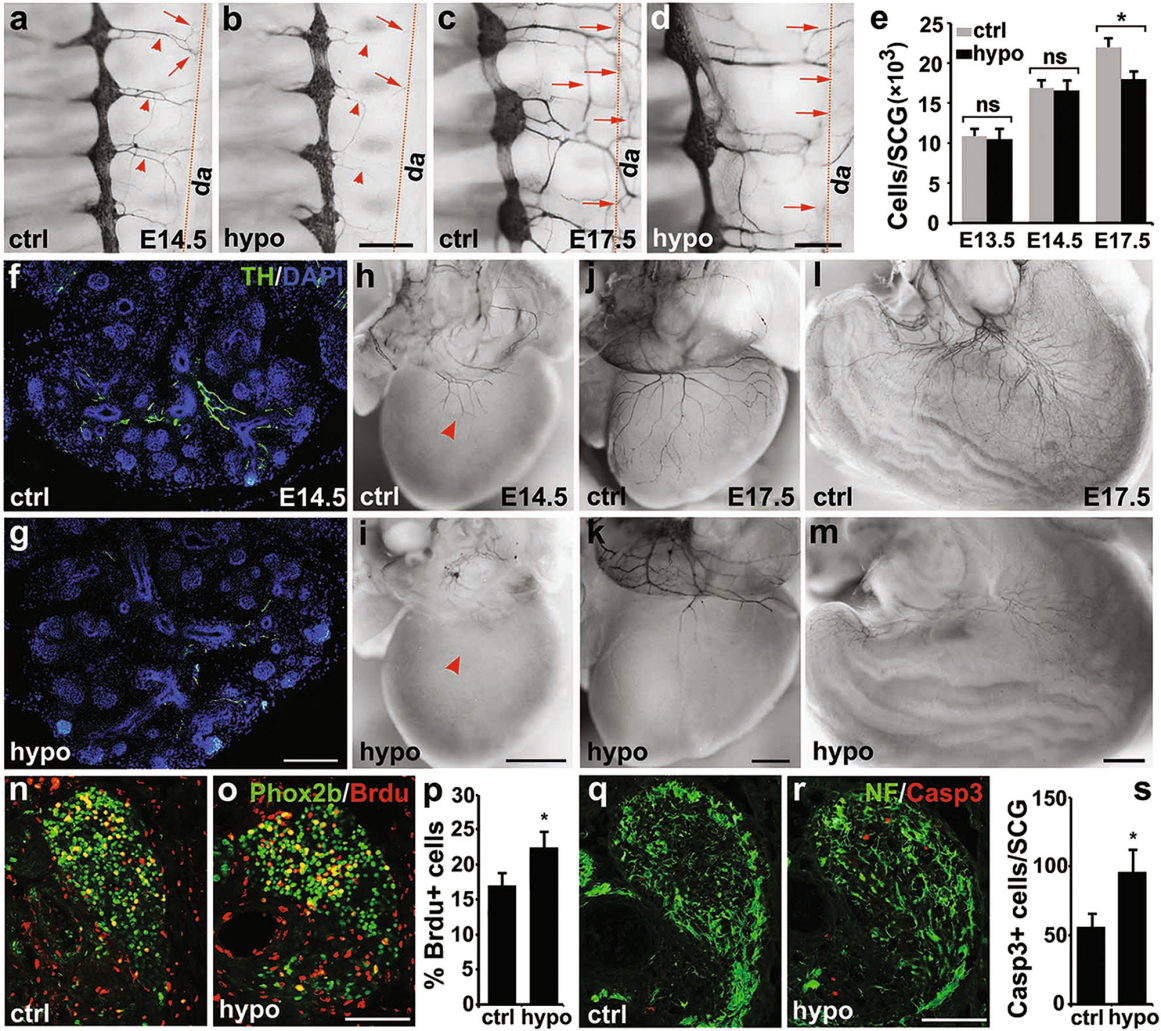
Impaired axon growth of sympathetic neurons in *Isl1* hypomorphic mutants. **a**–**d** Wholemount TH immunostaining of hypomorphic (hypo, *Isl1*^*f-neo/f-neo*^) and wildtype control (ctrl, *Isl1*^*+/+*^) embryos at E14.5 and E17.5 showing sympathetic ganglia and axons (arrowhead) extending toward, and neural plexus (arrow) around the dorsal aorta (da, red dashed line). Scale bar, 500 µm. **e** Quantitative analysis showing that the number of sympathetic neurons in *Isl1* hypomorphic embryos was comparable with control littermates at E13.5 and E14.5, but significantly reduced at E17.5. Error bars represent the s.d., *n* = 4, E13.5 (*p* = 0.854), E14.5 (*p* = 0.525), E17.5 (*p* = 0.014), two-tailed *t*-test. **f**–**m** Markedly reduced sympathetic innervations to the salivary gland (**f**, **g**), heart (**h**–**k**), and stomach (**l**, **m**) in *Isl1* hypomorphic embryos at E14.5 and E17.5 compared with control littermates revealed by TH immunostaining. Scale bar, 200 µm (**f**, **g**), 1 mm (**h**–**m**). **n**–**p** Increased proliferation in E14.5 *Isl1* hypomorphic sympathetic ganglia compared with controls revealed by co-immunostaining for BrdU and Phox2b. Error bars represent the s.d., *n* = 4, *p* = 0.011, two-tailed *t*-test. Scale bar, 100 µm. **q–s** Increased neuronal death revealed by co-immunostaining for activated caspase-3 and neurofilament (NF) in E14.5 *Isl1* hypomorphic sympathetic ganglia compared with controls. Error bars represent the s.d., *n* = 4, *p* = 0.024, two-tailed *t*-test. Scale bar, 100 µm. * *p* < 0.05, or ** *p* < 0.01

Marked cell loss in *Isl1* CKO sympathetic ganglia occurred well before robust sympathetic neuronal differentiation, precluding the study of the role of ISL1 during later sympathetic neuronal development. Therefore, we analyzed a mouse line with reduced *Isl1* expression (hypomorphic)^18^ (Supplementary Figure 2a-e). TH immunostaining revealed that the size of sympathetic ganglia and the number of neurons in *Isl1* hypomorphic embryos were not significantly altered prior to E17.5, although a reduction in sympathetic neuron number was observed in *Isl1* hypomorphic mice at E17.5 (Fig. 5a–e). This allowed us to examine the role of ISL1 during later developmental stages.

In control embryos, neurites of sympathetic ganglia began to extend branches to innervate the dorsa aorta (da, dashed line) at E14.5, and formed a complex nerve plexus around the dorsal aorta at E17.5 (Fig. 5a, c, arrow). In *Isl1* hypomorphic embryos, neurites of sympathetic ganglia appeared to be shorter with fewer branches at E14.5, and failed to form the aortic plexus at E17.5 (Fig. 5b, d, arrow). Similarly, sympathetic innervation to other target organs such as the salivary gland, heart, and stomach were markedly reduced in *Isl1* hypomorphic embryos at E14.5 and E17.5 (Fig. 5f–m).

At E14.5–16.5, we observed significantly increased neuronal proliferation (BrdU+/Phox2b+) in *Isl1* hypomorphic sympathetic ganglia (Fig. 5n–p). However, at E12.5–13.5, proliferation of sympathetic neurons was comparable between *Isl1* hypomorphic and control mice (Supplementary Figure 3), suggesting delayed cell cycle withdrawal of *Isl1* hypomorphic sympathetic neurons during later development. In addition, we observed significantly increased cell death, which may account for the overall loss of sympathetic neurons in *Isl1* hypomorphic mutants (Fig. 5q–s).

### RNA-seq analysis revealed dysregulation of genes important for axon growth and neurotransmission in *Isl1* hypomorphic sympathetic neurons during later development

**Fig. 6:**
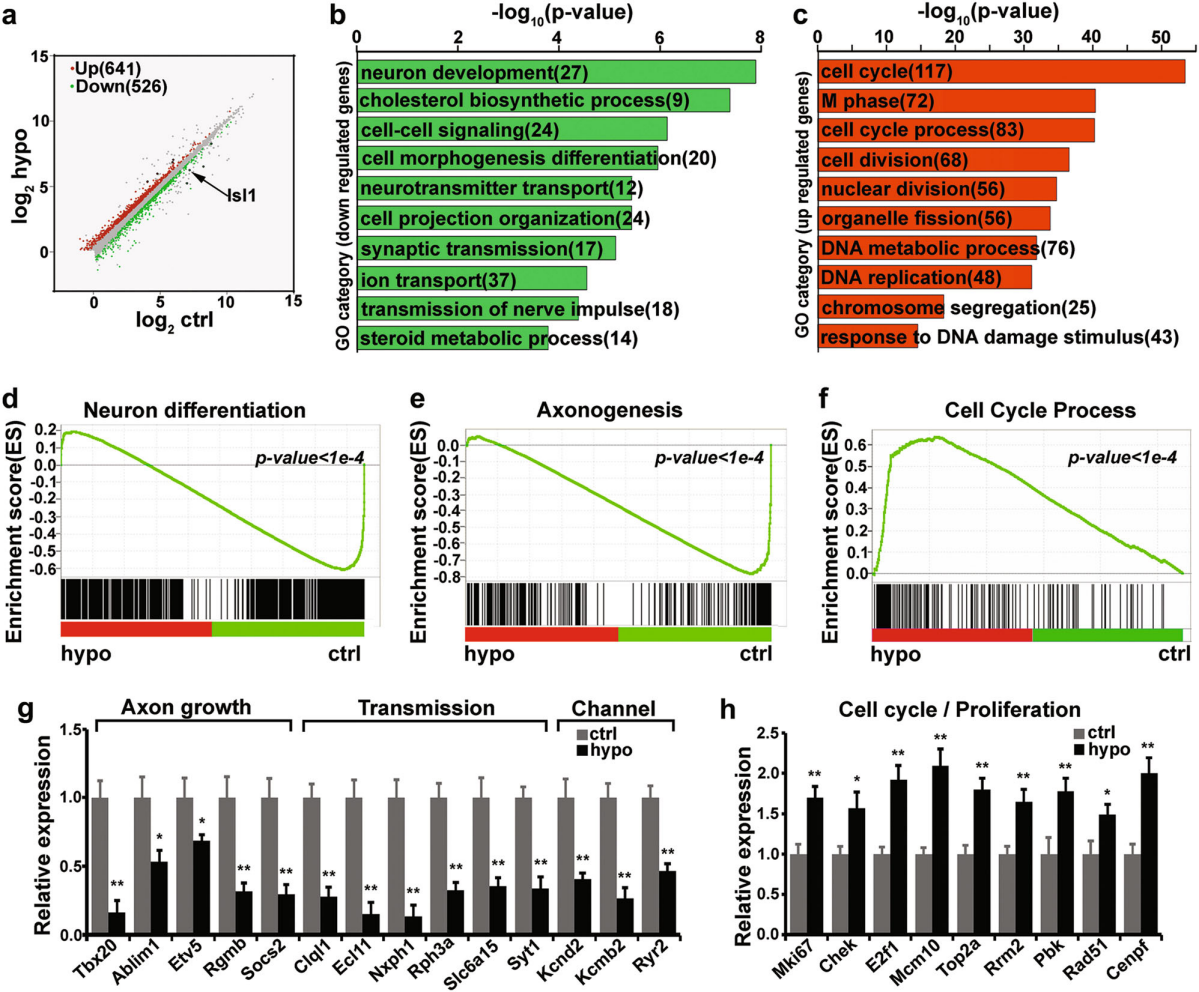
RNA-seq analyses reveal dysregulation of genes important for sympathetic neuron differentiation in *Isl1* hypomorphic mutants. **a** Scatterplot illustrating the relative gene expression of polyA-selected RNA transcripts from RNA-seq comparison of wildtype control and *Isl1* hypomorphic mutant sympathetic ganglia. Genes upregulated (641) or downregulated (526) (*p* < 0.05) in *Isl1* hypomorphic sympathetic neurons are shown in red and green, respectively. **b**, **c** GO terms enriched for genes upregulated or downregulated in *Isl1* hypomorphic sympathetic ganglia. **d**–**f** GSEA showing that gene sets representing neuron differentiation and axonogenesis are downregulated, whereas a gene set representing the cell cycle are significantly upregulated in *Isl1* hypomorphic sympathetic ganglia. Normalized enrichment scores (NESs) and *p*-values are shown. **g**, **h** qPCR validation of selected genes involved in axon growth, neurotransmission and ion channels, and the cell cycle in sympathetic neurons of E14.5 control and *Isl1* hypomorphic embryos. Error bars represent the s.d., *n* = 3, two-tailed *t*-test. * *p* < 0.05, or ** *p* < 0.01

We performed RNA-seq analysis on *Isl1* hypomorphic and wildtype sympathetic ganglia at E14.5 and identified 641 upregulated and 526 downregulated genes (*p < *0.05) (Fig. 6a and Supplementary Table 2). GO analysis revealed that downregulated genes were over-represented in categories such as neuron development and neurotransmission, whereas upregulated genes were mainly cell cycle related (Fig. 6b, c; Supplementary Table 2). Similarly, GSEA analysis revealed downregulation of gene sets representing neuron differentiation and axonogenesis, but upregulation of a gene set representing the cell cycle in *Isl1* hypomorphic embryos (Fig. 6d–f). Selected genes involved in axon growth, neurotransmission, and the cell cycle were verified by qPCR (Fig. 6g, h).

Intersection of the two RNA-seq data sets from *Isl1* CKO and hypomorphic mutants identified only 84 genes commonly regulated by ISL1 at early (9.2% overlap) and later (7.1%) developmental stages (Supplementary Figure 4). The commonly downregulated genes (46) were over-represented in categories such as protein secretion and synaptic vesicle transport, and upregulated genes (38) were involved in regulation of apoptosis (Supplementary Figure 4). Consistent with observed phenotypes, a subset of cell cycle-related genes were activated by ISL1 at early developmental stages but failed to downregulate in *Isl1* hypomorphic sympathetic neurons at later developmental stages. Another subset of genes involved in protein secretion and nervous system development were repressed early but activated later by ISL1 during sympathetic neuron development (Supplementary Figure 4). These results suggested a distinct temporal requirement for ISL1 during sympathetic neuron development.

### ISL1 is required for diversification of noradrenergic and cholinergic sympathetic neurons

**Fig. 7:**
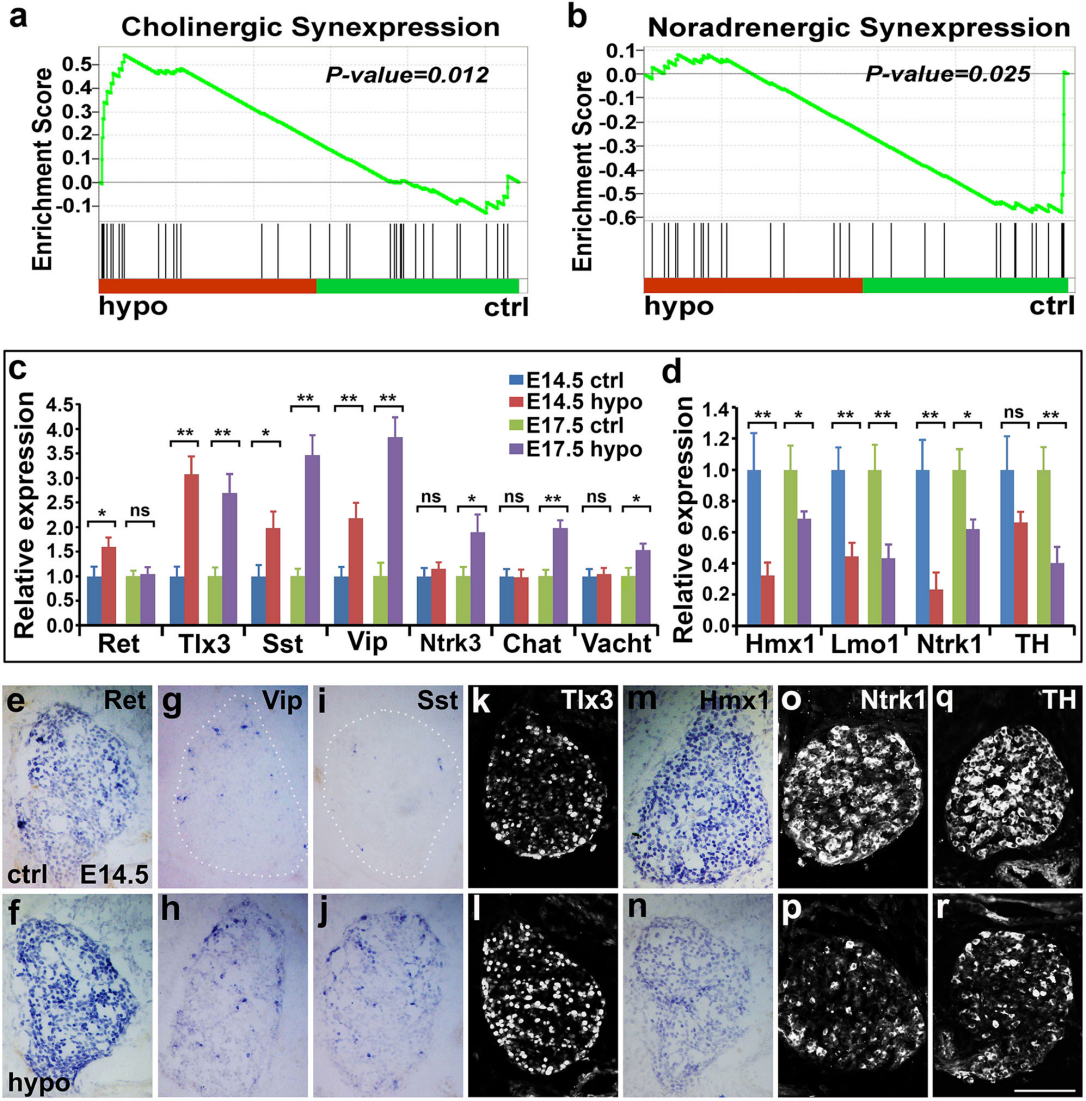
Decreased noradrenergic and increased cholinergic traits in *Isl1* hypomorphic mutant embryos. **a**, **b** GSEA showing downregulation of a gene set representing noradrenergic differentiation, but upregulation of a gene set representing cholinergic differentiation in *Isl1* hypomorphic sympathetic ganglia. Normalized enrichment scores (NESs) and *p*-values are shown. **c**, **d** qPCR showing relative mRNA expression of noradrenergic and cholinergic neuron genes in *Isl1* hypomorphic and control sympathetic ganglia at E14.5 and E17.5. Error bars represent the s.d., *n* = 3, two-tailed *t*-test. **e**–**r** Verification of selected genes associated with cholinergic neurons (*Ret, Vip, Sst, Tlx3*) and noradrenergic neurons (*Hmx1, Ntrk1, TH*) by in situ hybridization (**e**–**j**, **m**, **n**) and immunostaining (**k**, **l**, **o**–**r**) in E14.5 control and *Isl1* hypomorphic sympathetic ganglia. Scale bar, 100 µm, ns = not significant, * *p* < 0.05, or ** *p* < 0.01

RNA-seq and GSEA analyses revealed downregulation of a gene set representing noradrenergic differentiation, but upregulation of a gene set representing cholinergic differentiation in *Isl1* hypomorphic sympathetic neurons (Fig. 7a, b; Supplementary Table 3). These included cholinergic genes (*Ret, Tlx3, Sst*, and *Vip*) and noradrenergic genes (*Hmx1, Lmo1*, *Ntrk1*, and *TH*)^19^. Alterations in their expression were confirmed by qPCR, in situ and immunostaining (Fig. 7c–r). At E14.5 and E17.5, *Tlx3, Vip*, and *Sst* were upregulated, but *Hmx1, Lmo1*, and *Ntrk1* were downregulated in *Isl1* hypomorphic sympathetic ganglia. Ret expression was transiently upregulated at E14.5, but back to normal level at E17.5, whereas expression of *Ntrk3, Chat*, and *Vacht* was relatively normal at E14.5 but became upregulated at E17.5. Conversely, *TH* expression was unchanged at E14.5, but downregulated at E17.5. These data suggested that reduced ISL1 led to a failure both in repression of a cholinergic gene program and in maintenance of a noradrenergic program.

### ISL1 directly regulates a number of genes required for sympathetic development

**Fig. 8:**
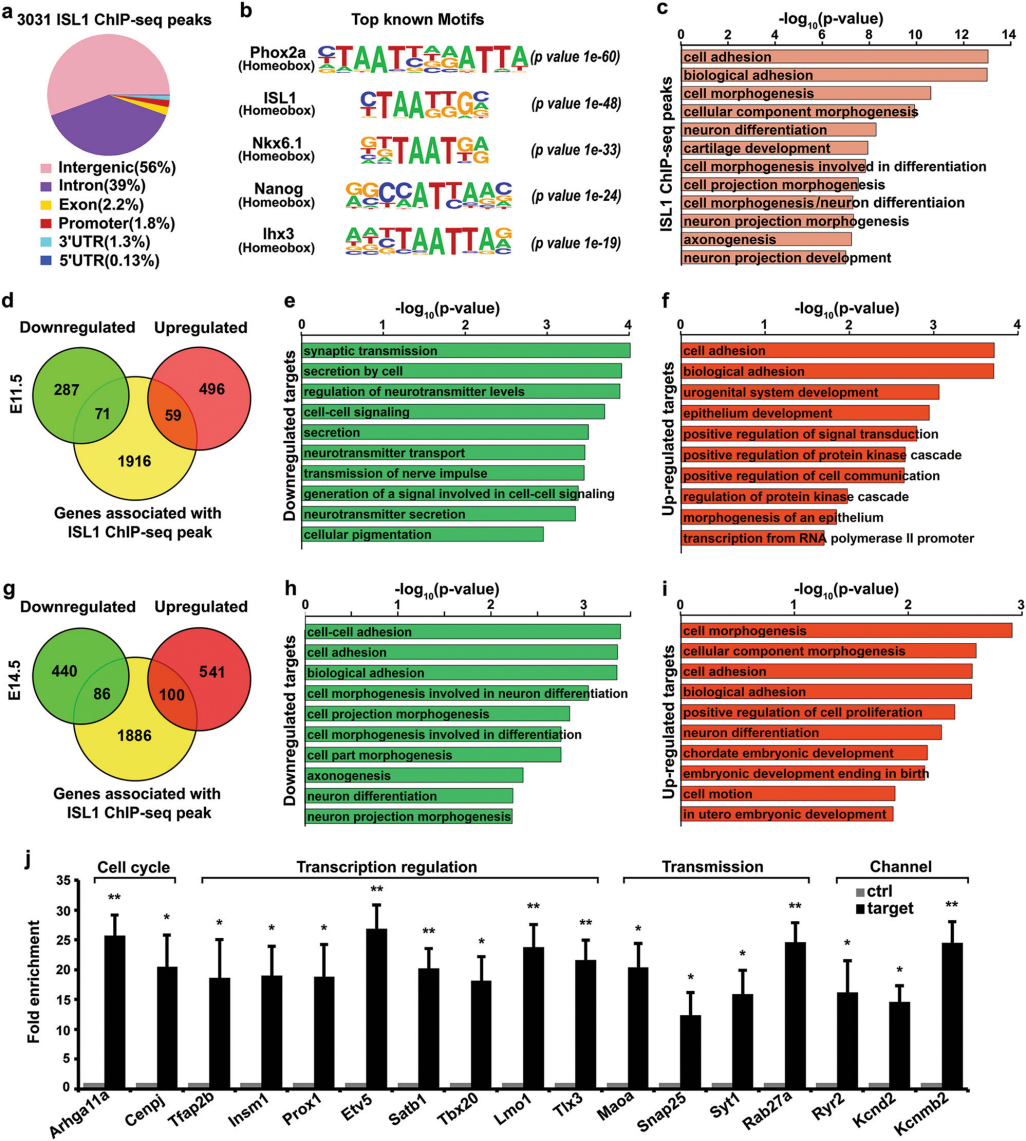
ISL1 directly regulates a number of genes required for sympathetic neuron development. **a** ChIP-seq ISL1-binding regions were mapped relative to their nearest transcription start site (TSS). Annotation includes whether a peak is in the TSS, transcription termination site, exon, 5ʹUTR, 3ʹUTR, intronic, or intergenic. **b** Top motifs enriched in the vicinity of ISL1-binding sites. **c** GO functional clustering of genes associated with ISL1 ChIP-seq peaks. **d** Overlay of ChIP-seq data and RNA-seq results from E11.5 *Isl1* CKO sympathetic ganglia revealed 71 downregulated and 59 upregulated genes as potential direct targets of ISL1 in sympathetic neurons; *p* = 1.56 × 10^−7^, Fisher’s exact test. **e**, **f** GO functional clustering of these genes bound by ISL1 and downregulated (**e**) or upregulated (**f**) in E11.5 *Isl1* CKO sympathetic neurons. **g** Intersection of ChIP-seq data and RNA-seq results from E14.5 *Isl1* hypomorphic sympathetic ganglia revealed 86 downregulated and 100 upregulated genes as potential direct targets of ISL1 in sympathetic neurons; *p* = 4.6 × 10^−13^, Fisher’s exact test. **h**, **i** GO functional clustering of these genes bound by ISL1 and dysregulated in E14.5 *Isl1* hypomorphic sympathetic neurons. **j** ChIP-qPCR validation of ISL1 binding at selected targets in E12.5–E14.5 sympathetic neurons, including those genes involved in the cell cycle, transcription regulation, neurotransmission, and ion channels. Error bars represent the s.d., *n* = 3, two-tailed *t*-test. *p < 0.05, or ** *p* < 0.01

To gain an insight into the direct downstream targets of ISL1 in sympathetic neurons, we performed ISL1 ChIP-seq analyses on E12.5–14.5 sympathetic ganglia. Analyses of these data revealed 3031 ChIP-seq peaks for ISL1, which also contained other motifs for Phox2a, Nkx6.1, Lhx3, Nanog, and Gata3 (Fig. 8a, b; Supplementary Table 4 and 5). Spatial annotation of ISL1 binding sites revealed 2046 potential ISL1 targets, including those involved in neuron differentiation and axonogenesis (Fig. 8c, Supplementary Figure 5a and Table 4). Intersection of ChIP-seq data with RNA-seq data from *Isl1* CKO and hypomorphic mutants revealed 130 (71 downregulated, 59 upregulated) and 186 (86 downregulated, 100 upregulated) genes that were directly regulated by ISL1 during early and later sympathetic development, respectively (Fig. 8d, g; Supplementary Table 6 and 7). For direct targets of ISL1 downregulated in E12 *Isl1* CKO mutants, over-represented categories included neurotransmission and cell–cell signaling, whereas upregulated targets of ISL1 were involved in, e.g., cell adhesion and signal transduction (Fig. 8e, f; Supplementary Table 6). Similarly, for direct targets of ISL1 downregulated in E14.5 *Isl1* hypomorphic mutants, over-represented categories included neuron differentiation and axonogenesis. For upregulated targets of ISL1, categories such as cell morphogenesis, neuron differentiation, and cell proliferation were prominent (Fig. 8h, i; Supplementary Table 7).

A close examination of direct targets of ISL1 downregulated in *Isl1* CKO revealed a number of genes essential for sympathetic neuron development (e.g., *Gata3, Insm1, Tlx3, Lin28b*), axon growth, and neurotransmission (e.g., *Bmpr1b, Plxna4, Snap25*). Similarly, many direct targets of ISL1 downregulated in *Isl1* hypomorphic sympathetic ganglia were involved in neuronal differentiation (e.g., *Tbx20, Etv5, Phox2a*) and neurotransmission (e.g., *Ryr2, Kcnd2, Kcnmb2*). Intersection of direct targets of ISL1 found in *Isl1* CKO and hypomorphic mutants revealed 41 common targets of ISL1, many of which, e.g., *Lmo1, Egln3, Tbx20*, were positively regulated by ISL1 at both early and late stages (Supplementary Table 8). However, several direct targets of ISL1, such as *Prox1, Tlx3*, and several cell cycle genes, were downregulated in *Isl1* CKO during early development, but upregulated in *Isl1* hypomorphic mutants during later development (Supplementary Table 8). Selected targets of ISL1 were verified by ChIP-qPCR, and genome browser views of representative common targets of ISL1 were shown (Fig. 8j and Supplementary Figure 5b).

Notably, a number of targets of ISL1 downregulated in *Isl1* CKO mutants were implicated in neuroblastoma pathogenesis^20–23^, including *Lmo1, Gata3, Prox1, Lin28b*, and *Alk*. We found that ISL1 binds to an evolutionary conserved region within the first intron of *Lmo1* (mm9:chr7:116289070-116289656) in mouse sympathetic neurons that, by whole-genome alignment, overlaps with the super-enhancer of LMO1 identified in human neuroblastoma^20^.

## Discussion

### Distinct temporal requirements for ISL1 in sympathetic neuron proliferation and differentiation during sympathetic neuron development

Sympathetic neurons continue to proliferate after the acquisition of pan-neuronal and noradrenergic properties^5^. From E14.5 onward, a majority of sympathetic neurons undergo cell cycle withdrawal and terminal differentiation and maturation; however, the mechanisms underlying these temporal events remain largely unknown. At early developmental stages, ablation of *Isl1* resulted in reduced sympathetic neuron proliferation and reduced expression of cell cycle genes, including *Ccnd1* and *Foxm1*. Expression of a number of transcription factors critical for sympathetic neuron proliferation was also downregulated in *Isl1* CKO mutants^24–27^, including Gata3, Insm1, and Prox1. Although mechanisms by which these transcription factors control sympathetic neuron proliferation are unclear, these data suggest that ISL1 may regulate sympathetic neuron proliferation by controlling cell cycle gene expression.

Differentiation of noradrenergic and pan-neuronal phenotypes is controlled by an early gene regulatory network^3^, including *Mash1*, *Phox2b/2a*, *Hand2*, *Gata2/3*, and *Insm1*. We found that ISL1 is downstream of Phox2b, but upstream of Gata2/3, Insm1/2, and Hand1. ISL1 acts parallel to Hand2, since Hand2 expression is not changed in *Isl1* CKO mutants and vice versa^28^. ISL1 is dispensable for initial noradrenergic differentiation, since expression of TH and DBH is not altered in *Isl1* CKO mutants. But instead, ISL1 directly or indirectly regulates sets of genes essential for sympathetic neuron development, axon growth, and neurotransmission, suggesting that ISL1 is essential for generic sympathetic neuronal differentiation during early sympathetic neuron development.

Reduced *Isl1* expression in *Isl1* hypomorphic mice caused impaired sympathetic innervations, delayed cell cycle withdrawal, and reduced expression of genes involved in axon growth and neurotransmission, demonstrating a continued requirement for ISL1 in sympathetic neuron differentiation and maturation at later development stages. Analyses of ChIP-seq and RNA-seq data sets from both Isl1 CKO and hypomorphic mutants revealed ISL1 regulates common, yet distinct gene programs during early and later sympathetic neuronogenesis. *Tlx3, Prox1*, and several cell cycle-related genes were among the direct targets of ISL1 that were downregulated in *Isl1* CKO but upregulated in *Isl1* hypomorphic mutants, suggesting that ISL1 may act both as an activator and a repressor in a context-dependent manner during sympathetic neuron development. Interestingly, a comparison of ISL1 targets in different cell types (sympathetic neurons, cardiac progenitors^29^, and sinus node pacemakers^30^) revealed few, if any, common targets that could account for the observed phenotypes in each of these mutants, suggesting that the action of ISL1 is cellular context-dependent.

Molecular mechanisms of ISL1 in sympathetic neurons remain unknown. Interestingly, ablation of Isl1 causes downregulation of a number of epigenetic regulators^29,31,32^ (Dnmt1, Kdm6a, and Satb1) critical for neuronal development. In the heart, ISL1 physically interacts with Kdm6a to promote H3K27me3 demethylation at the enhancers of target genes encoding key cardiac transcription factors^29^. It is tempting to speculate that ISL1 might regulate the expression of sympathetic developmental genes via epigenetic mechanisms.

### Essential role of ISL1 in coordination of initial neurogenesis and gliogenesis by modulating BMP and Notch signaling pathways

BMP and Notch pathways are essential for coordinating progenitor maintenance and neuronal differentiation during sympathetic nneurogenesis^6^. Ablation of *Isl1* resulted in increased Notch signaling but decreased BMP signaling. Accordingly, genes associated with NC and glial progenitors were significantly upregulated, suggesting that during initial sympathetic neurogenesis, ISL1 is required for coordination of lineage differentiation of sympathetic neurons and glia by modulating BMP and Notch pathways. Interestingly, previous studies have revealed a role of ISL1 in cell fate specification and determination in sensory neurons of the dorsal root ganglion and spinal motor neurons. Ablation of *Isl1* in NC cells leads to reduced expression of genes essential for nociceptive neuron differentiation, but a failure in developmental downregulation of genes characteristic of sensory progenitors^15^. In spinal motor neurons, reduced *Isl1* expression leads to conversion of motor neurons to V2 interneurons, although underlying mechanism remains unknown^18^.

### ISL1 is required for diversification of noradrenergic and cholinergic sympathetic neurons

Segregation of initially bimodal sympathetic neurons into noradrenergic and cholinergic neurons occurs later during development and involves antagonistic actions between pro-cholinergic (*Ntrk3*/Ret/Tlx3) and pro-noradrenergic (*Hmx1*/*Ntrk1*) factors^13,14^. In *Isl1* hypomorphic sympathetic ganglia, we observed significantly reduced expression of genes essential for noradrenergic differentiation (e.g., *Hmx1, Lmo1, Ntrk1*, *TH*), but increased expression of genes essential for cholinergic differentiation (*Ret, Tlx3, Sst, Vip, Ntrk3, Chat*, *Vacht*), demonstrating that ISL1 is required for repression of cholinergic characteristics and maintenance of noradrenergic characteristics during later developmental stages. Previous studies have shown that, in forebrain and spinal cholinergic motor neurons, ISL1, complexed with LHX3 or LHX8, directly binds to and activates the expression of genes of cholinergic traits^33^. However, we did not find direct binding of ISL1 to the enhancers/promoters of *Chat*, *Vacht*, or other cholinergic genes.

### ISL1 and neuroblastoma

Neuroblastoma is an aggressive pediatric tumor arising from developing sympathetic neurons. ISL1 is overexpressed in most types of neuroblastoma, particularly in poorly differentiated neuroblastomas that indicates a poor prognosis^34,35^. ISL1 is also a minimal residual disease marker of neuroblastoma^34^. However, the role of ISL1 in neuroblastoma tumorigenesis is unknown. We found that a number of genes implicated in neuroblastoma pathogenesis are downstream of ISL1 (e.g., *Lmo1, Ccnd1*, *Tfap2b*, *Prox1*, *Casz1*, *Foxm1*, *Lin28b*, *Ccnd1*, and *Alk*)^20–23,34,36–38^, many of which are direct targets of ISL1. Notably, LMO1 is a neuroblastoma oncogene associated with more proliferative and aggressive phenotype^39^. A polymorphism within a super-enhancer in the first intron of LMO1 that preserves an evolutionarily conserved GATA factor binding motif predisposes to neuroblastoma. Knockdown of GATA3 results in decreased LMO1 expression and suppression of neuroblastoma cell growth^20^. We found that ISL1 binds to an evolutionary conserved region within the first intron of *Lmo1* (chr7:116289070-116289656) in mouse sympathetic neurons that overlaps with the super-enhancer of LMO1 identified in human neuroblastoma, suggesting co-regulation of LMO1 expression by ISL1 and GATA3 and a potential role of ISL1 in controlling neuroblastoma cell proliferation.

Taken together, our study uncovered a temporal requirement for ISL1 during sympathetic neurogenesis (Supplementary Figure 6), and implicated *Isl1* as a candidate gene for neuroblastoma.

## Materials and methods

### Transgenic mice

Generation of *Wnt1-Cre*;*Isl1*^f/f^ and *Isl1* hypomorphic mice(*Isl1*^*f-neo/f-neo*^) have been reported previously^18^. To facilitate the visualization and lineage tracing of sympathetic neurons, *Isl1*^f/f^ mice were crossed onto a *Rosa26-LacZ* or -*Tomato* reporter background^40^. All the experiments involving mice were carried out in accordance with protocols approved by the Animal Committee of Tongji University School of Medicine (TJmed-010-10).

### In situ hybridization, immunostaining, and X-gal staining

In situ hybridization, immunostaining, and X-gal staining were performed as described^30^. Fragments amplified from mouse cDNA were used to generate the RNA probes for *Insm2, Tlx3, Tfap2b, Hand1*, and *Lmo1*. Riboprobe for mouse *Vip, Sst*, and *Ret* were kindly provided by Dr. Leping Cheng (Chinese Academy of Sciences). For BrdU staining, pregnant mice were injected with 300 μl of BrdU (100 μg/g body weight, Sigma). For cell counting, sections of sympathetic ganglia of the appropriate developmental stages were cut at 10 μm, every fourth section was stained, and positive cells were counted. For proliferation, the total number of BrdU+ sympathetic neurons (Phox2b+) were counted and expressed as percentage of total Phox2b+ neurons. For cell death assay, the total number of Casp-3+ neurons (Tomato+) were counted and expressed as percentage of total Tomato+ neurons. At least 5–8 matched sections were analyzed and three samples per genotype per time point were analyzed. Detailed information of antibodies is available in the Supplementary material.

### Sympathetic ganglion dissection and culture

Sympathetic ganglia were dissected from E11.5–12 embryos of genotype *Wnt1-Cre;Isl1*^*f/f*^ (CKO) and *Wnt1-Cre;Isl1*^*+/+*^ (ctrl) with *Rosa26-tdTomato* reporter background. SCGs of E12 *Isl1* CKO and control embryos on *Rosa-tdTomato* background were cultured in matrigel (BD Biosciences) in media containing NGF (20 ng/ml) and NT3 (20 ng/ml). Detailed protocol of sympathetic ganglion dissection and culture are available in the Supplementary material.

### RNA-seq and ChIP-seq data analysis

RNA-seq analyses with two biological repeats were performed as described^30^. ChIP-seq was performed as previously described^41^. The complete RNA-seq and ChIP-seq data sets are available from the Gene Expressing Omnibus database (http://www.ncbi.nlm.nih.gov/geo/) under the accession number GSE93308. For experimental and data analysis details, see the Online Data Supplement.

### Statistical analysis

All experiments were performed for at least three independent times and respective data were used for statistical analyses. Data were presented as mean ± s.d., and a two-tailed *t*-test was used for two-group comparisons. Differences were considered statistically significant at a value of *p* ≤ 0.05*, *p* ≤ 0.01**. For experimental details, see the online Supplementary material.

## Electronic supplementary material

Supplementary methods, Figure and Table Legends

Supplementary figure 1

Supplementary figure 2

Supplementary figure 3

Supplementary figure 4

Supplementary figure 5

Supplementary figure 6

Supplementary Table 1

Supplementary Table 2

Supplementary Table 3

Supplementary Table 4

Supplementary Table 5

Supplementary Table 6

Supplementary Table 7

Supplementary Table 8

Supplementary Table 9
