# Supplementary material for: Temporal requirements for ISL1 in sympathetic neuron proliferation, differentiation, and diversification
Source: Cell Death Dis. 2018 Feb 14;9(2):247. doi: 10.1038/s41419-018-0283-9 (PMC5833373; doi:10.1038/s41419-018-0283-9)
Supplement: Supplementary file 1 — Supplementary methods, Figure and Table Legends [file 41419_2018_283_MOESM1_ESM.docx]

**Supplementary Information**

**Antibodies**

The following primary antibodies were used: mouse anti-ISL1/2 (39.4D5, DSHB), rabbit anti-ISL1 (ab20670, Abcam), rabbit anti-ISL1 (ab109517, Abcam), rat anti-BrdU (ab6326, Abcam), rabbit anti-TH (ab152, Millipore), rabbit anti-Casp-3 (9662, Cell Signal), goat anti-Sox10 (sc-17343, Santa Cruz Biotechnology Inc), rabbit anti-MKi67 (ab16667, Abcam), rabbit anti-Ccnd1 (ab16663, Abcam), rabbit anti-Tlx3 (a generous gift from Dr. Leping Cheng), rabbit anti-TrkA (Ntrk1) and rabbit anti-Phox2b (a generous gift from Dr. Brunet JF), mouse anti-NF (2H3, DSHB) and rabbit anti-Prox1 (ab38692, Abcam). The secondary antibodies were Alexa 488 or 594 conjugated (Invitrogen).

**Sympathetic ganglion dissection**

Sympathetic ganglia were dissected from E11.5-12 embryos of genotype *Wnt1-Cre;Isl1^f/f^* (CKO) and *Wnt1-Cre;Isl1^+/+^* (ctrl) with *Rosa26-tdTomato* reporter background. The presence of *tdTomato* reporter allele allowed embryos expressing Wnt1-Cre to be visually genotyped under the fluorescence microscope and only red fluorescent (*Tomato*+) embryos were used for dissection. To dissect *Isl1* CKO mutants, *Tomato*+ embryos were kept individually in 24-well plate and pooled after genotyping. Embryos were cut open the chest and abdominal cavity, and the internal organs were removed under a dissecting microscope to expose the sympathetic ganglia/chain (including superior cervical ganglia and stellate ganglia). The sympathetic chain or individual ganglion was carefully dissected under the fluorescence microscope and collected individually in a 24-well plate with DEPC-PBS. To our experience, E11.5-12 are the earliest stages at which sympathetic ganglia/chain are clearly visible (red fluorescent) and can be dissected under the fluorescence microscope. The ganglia were transferred to a 35 cm dish with DEPC-PBS, re-examined under the fluorescence microscope, and contaminating tissues (non-fluorescent) were cleaned. Similarly, sympathetic ganglia from E14.5 and E17.5 wildtype embryos were dissected under a dissecting microscope and collected individually in a 24-well plate with DEPC-PBS, and pooled after genotyping.

**Sympathetic ganglion culture**

SCGs of E12 *Isl1* CKO and control embryos on Rosa-tdTomato background were cultured in matrigel (BD Biosciences) in media containing NGF (20 ng/ml) and NT3 (20 ng/ml) for 48 hours. Neurite outgrowth in SCG explants was quantified by measuring the area covered by the axons of each explant relative to the area occupied by the cell bodies.

**RNA isolation and qPCR analysis**

Sympathetic ganglia were dissected from control and *Isl1* mutant embryos at desired stages. Samples from 10-20 embryos of the same genotype were pooled at the time of RNA extraction. For the qPCR analysis of E11.5-12 samples, sympathetic ganglia were digested and FACS-sorted. RNA isolation was performed using the RNeasy Micro kit (Qiagen) following the manufacturer’s instructions. cDNA synthesis was performed using SuperScript™ II Reverse Transcriptase Kit (Invitrogen, 18064014) and random primers. qPCR was performed using Power SYBR Green PCR Master Mix (Cat # 4367659, Life Technologies) and primers as listed (Supplementary Table 9). Mouse GAPDH and 18sRNA were used as internal reference genes. Relative transcript expression of control and mutant samples was calculated by the 2**^ΔΔCt^** method. Statistical significance was assessed using the 2-tailed *t* test with at least three biological replicates for each genotype.

**RNA-seq and data analysis**

RNA-seq analyses with two biological repeats were performed as described ^[1](#_ENREF_1)^. The complete RNA-seq datasets are available from the Gene Expressing Omnibus database (http://www.ncbi.nlm.nih.gov/geo/) under the accession number GSE93308. Briefly, total RNA (100 ng) was incubated with Oligo(dT) magnetic beads to isolated mRNA. RNA-seq libraries were prepared with the SMARTer cDNA library construction kit (Clontech) according to manufacturer’s instructions and sequenced using Illumina HiSeqTM 2000 with paired-end sequencing at 90-bp read length (BGI). Sequencing reads were aligned to the mm9 reference genome using TopHat version 2.3.0 with default parameter setting. The uniquely mapped reads were assembled into transcripts guided by mouse reference annotation with cuffdiff version 2.2.1 to determine transcript expression values, which were normalized by FPKM (fragments per kilobase per million). The differentially expressed genes were identified by FPKM ≥ 1 in either one of the two conditions, | the change of FPKM | ≥ 1.5 fold and the FDR less than 0.05 (*Isl1* CKO), or *p* < 0.05 (*Isl1* hypomorphic), which were estimated by cuffdiff. GO enrichment analysis were performed by DAVID (v6.7) gene annotation tool.

**ChIP-seq data analysis**

ChIP-seq was performed as previously described[^2^](#_ENREF_2). Briefly, sympathetic ganglia from about 40 wildtype embryos at E12.5-14.5 were digested into single cell suspension with a mixture of collagenase II (1 mg/ml) and trypsin (0.1%). Cells were cross-linked with 1% formaldehyde for 10 minutes at room temperature, and then quenched with 0.125 mM glycine. 1-5×10^5^ cross-linked cells were lysed in 0.5% SDS buffer. 100-300bp chromatin fragments were obtained after sonication with indicated condition. 5% total chromatin fragments were kept as Input. Dilute the chromatin fragments 1:5 with Sonication Equilibration Buffer (10 mM Tris-HCl, 140mM NaCl, 0.1 % Sodium Deoxycholate, 1% Triton X-100, 1 mM EDTA and 1X Protease Inhibitors EDTA free (Roche)). To immobilize the chromatin on magnetic beads, 15µl of Dynabeads Protein G coated with Histone H3 antibody (ab1791) were added to the diluted chromatin extracts and incubated for 20 hours at 4°C. Bead bound chromatins were washed 3 times with 150 µl of 10 mM Tris-HCl + 1X Protease Inhibitors EDTA free and suspended in 20 µl of the same buffer. Sequencing libraries were prepared on beads with bound chromatin by blunting, A-tailing, adaptor ligation as previously described [^2^](#_ENREF_2) using Y-shapped Indexed Adaptors (containing P5 and P7 sequences). Beads with bound and indexed chromatin were washed and incubated in Chromatin Release Buffer (250mM NaCl, 1% SDS, 1% Sodium Deoxycholate, 1X protease Inhibitors) to release the indexed chromatin, and samples concentrated using a 50Kda cutoff Centricon (Amicon). The indexed chromatins were resuspended to a final volume of 250 µl Dilution Buffer (10mM Tris-HCl pH 8, 100mM NaCl, 1mM EDTA, 1X Protease Inhibitors EDTA free), and then 50 µl of ISL1 antibody (ab109517, Abcam) coated protein G magnetic Dynabeads (Invitrogen) were added into the chromatin samples and incubated for 8 hour at 4°C. The beads were extensively washed, then eluted in 50 µl Elution buffer (0.5% SDS, 300 mM NaCl, 5 mM EDTA, 10 mM Tris-HCl pH 8.0). The eluate was treated sequentially with RNaseA for 30 min at 37°C, Proteinase K for two hours at 37°C and 8 hours at 65°C to revert formaldehyde crosslinking. ISL1 ChIPed DNA was purified with phenol chloroform and PCR-amplified for 15 cycles using Q5 High-Fidelity DNA Polymerase (NEB, USA). The amplified libraries were sequenced with 75 bp single-end sequencing read using Illumina Next 500. About 27.12 and 46.02 million reads were obtained for ISL1 and input samples, respectively. Reads were aligned to the mouse reference genome (mm9) using Bowtie2 without mismatch. Only uniquely mapped reads were used to peak calling for transcription factor ISL1 through MACS version 1.4.2 20120305 with *p-value* cutoff for peak detection less than 1e-4. The annotation of peaks was performed with the annotate Peaks command from HOMER (http://homer.salk.edu/homer/) based on the nearest RefSeq transcription start sites (TSSs). Enriched de novo and HOMER known motifs of ISL1-binding peaks were identified by HOMER command findMotifsGenome.pl with default parameters. ISL1 ChIP-seq reads mapped around their summits were carried by annotatePeaks.pl with bin size in 5-bp, and corresponding heat map was ordered by the intensity of binding. The intersections of ISL1-binding peaks targeted genes and differentially expressed genes for ISL1 mutation at E12 and E14.5 were performed by R package “Venn Diagram”. The visualization of ChIP-seq data was generated using the Integrative Genome Viewer IGV 2.3 (<http://www.broadinstitute.org/igv/>). The complete ChIP-seq datasets are available from the GEO database under the accession number GSE93308.

ChIP-qPCR was performed as described [^1^](#_ENREF_1).ISL1 ChIP-DNA and IgG ChIP-DNA were used as templet and primers were listed in S10 Table. The data were normalized to the negative controls with primers to sequences that are adjacent (>1-5 kb) to ISL1 binding regions but do not contain ISL1 binding motifs. At least three independent samples were analyzed.

**Supplementary References**

1. Liang X, Zhang Q, Cattaneo P, Zhuang S, Gong X, Spann NJ*, et al.* Transcription factor ISL1 is essential for pacemaker development and function. *J Clin Invest* 2015, **125**(8)**:** 3256-3268.

2. Lara-Astiaso D, Weiner A, Lorenzo-Vivas E, Zaretsky I, Jaitin DA, David E*, et al.* Immunogenetics. Chromatin state dynamics during blood formation. *Science* 2014, **345**(6199)**:** 943-949.

**Supplementary Figure Legends**

Supplementary Figure 1. Reduced sympathetic innervation of the heart and altered neurotransmitter expression in *Isl1* CKO mutants. (**a**-**d**) Wholemount TH immunostaining showing reduced sympathetic innervation of *Isl1* CKO hearts compared with control hearts at E14.5 and E17.5, n=3. Scale bar, 500µm. (**e**, **f**) qPCR revealed decreased expression of *Chga, Vip* and *Vstm2l* but increased expression of *Pyy, Chgb* and *Srgn* in *Isl1* CKO sympathetic ganglia. Error bars represent the s.d. n=3. * *p<0.05*, or ** *p<0.01*. 2-tailed *t* test.

Supplementary Figure 2. Expression of ISL1 in hypomorphic sympathetic neuron. (**a**-**d**) Immunostaining demonstrated significantly reduced expression of ISL1 in hypomorphic SCG at E11.5 and E14.5. Scale bar, 100µm. Cranial ganglia/nerves X (X), superior cervical ganglion (SCG), carotid body (CB), cochlea (CCA). (**e**) qPCR analysis revealed reduced expression of ISL1 in hypomorphic sympathetic neuron at E14.5. Error bars represent the s.d. n=3, *p=0.0067*. The asterisks represent: * *p<0.05*, or ** *p<0.01*. 2-tailed *t* test.

Supplementary Figure 3. BrdU staining in *Isl1* hypomorphic sympathetic neuron at E12.5 and E13.5. (**a**-**d**) BrdU staining in the SCG at E12.5 and E13.5. Scale bar, 50µm. (**e**) Quantitative analysis revealed no significant change of proliferation in SCG between ISL1 hypomorphic mutant and control littermates at E12.5 and E13.5. Error bars represent the s.d. n=4,  E12.5 (*p=0.45)*, E13.5 (*p=0.35*), 2-tailed *t* test.

Supplementary Figure **4.** **Temporally distinct roles of ISL1 during sympathetic development.** Intersection of the two RNA-seq datasets from *Isl1* CKO and hypomorphic mutants suggesting common and distinct temporal requirements for ISL1 during sympathetic neuron development.

Supplementary Figure 5. ChIP-seq analysis on ISL1 in sympathetic neuron. (**a**) KEGG Pathway enriched for ISL1 direct target genes. (**b**) IGV genome browser view showing three ISL1 ChIP-seq detected enhancers at key ISL1 regulated genes.

Supplementary Figure 6. The regulatory network controlling sympathetic neuron development. (**a**) A scheme depicting roles and genetic interactions of factors essential for sympathetic neuron development during early developmental stages. (**b**) A schematic representation of gene-regulatory interactions determining noradrenergic and cholinergic sympathetic subtypes during late developmental stages. Arrows show positive regulation and red lines show negative regulation.

Supplementary Table **Legends**

Supplementary Table 1. RNA-Seq analysis of differential gene expression of sympathetic neurons between control and *Isl1* CKO mutants. (A) List of all genes expressed (RPKM≥1) in sympathetic neuron at E11.5. (B) List of genes (358) downregulated in *Isl1* CKO mutant sympathetic neuron at E11.5 (Fold Change mut vs ctrl≤-1.5). (C) List of genes (555) upregulated in *Isl1* CKO mutant sympathetic neuron at E11.5 (Fold Change mut vs ctrl≥1.5).

Supplementary Table 2. RNA-seq analysis of differential gene expression of sympathetic neuron between control and *Isl1* hypomorphic mutants. (A) List of all genes expressed (RPKM≥1) in sympathetic neuron at E14.5. (B) List of genes (526) downregulated in *Isl1* hypomorphic mutant sympathetic neuron at E14.5 (*p*≤*0.05*). (C) List of genes (641) upregulated in *Isl1* hypomorphic mutant sympathetic neuron at E14.5 (*p*≤*0.05*). (D) Gene Ontology analysis of genes downregulated in *Isl1* hypo mutant sympathetic neuron. (E) Gene Ontology analysis of genes upregulated in *Isl1* hypo mutant sympathetic neuron.

Supplementary Table 3. Noradrenergic and cholinergic neuron enriched genes. (A) Noradrenergic neuron enriched genes. (B) Cholinergic neuron enriched genes.

Supplementary Table 4. ChIP-seq analysis of direct targets of ISL1. (A) List of ISL1 ChIP-seq peaks significantly enriched over input (FDR threshold = 0.001; Fold over input required = 4.00). (B) Gene Ontology analysis of all genes associated with ISL1 ChIP-seq peaks.

Supplementary Table 5. **Homer known motif enrichment results**.

Supplementary Table 6. Gene Ontology analysis of direct targets of ISL1 at early sympathetic neuron developmental stage. (A) List of all genes (71) downregulated in *Isl1* CKO mutant sympathetic neuron, associated to ISL1 ChIP-seq peak. (B) List of all genes (59) upregulated in *Isl1* CKO mutant sympathetic neuron, associated to ISL1 ChIP-seq peak.(C) Gene Ontology analysis of all genes downregulated in *Isl1* CKO mutant sympathetic neuron, associated to ISL1 ChIP-seq peak.(D) Gene Ontology analysis of all genes upregulated in *Isl1* CKO mutant sympathetic neuron, associated to ISL1 ChIP-seq peak.

Supplementary Table 7. Gene Ontology analysis of direct targets of ISL1 at later sympathetic neuron developmental stage. (A) List of all genes downregulated (86) in *Isl1* hypomorphic mutant sympathetic neuron, associated to ISL1 ChIP-seq peak. (B) List of all genes (100) upregulated in *Isl1* hypomorphic mutant sympathetic neuron, associated to ISL1 ChIP-seq peak.(C) Gene Ontology analysis of all genes downregulated in *Isl1* hypomorphic mutant sympathetic neuron, associated to ISL1 ChIP-seq peak.(D) Gene Ontology analysis of all genes upregulated in *Isl1* hypomorphic mutant sympathetic neuron, associated to ISL1 ChIP-seq peak.

Supplementary Table 8**. List of common direct target genes in both E11.5 and E14.5 sympathetic neuron.**

Supplementary Table 9. Primers used in the study. (A) qRT-PCR primers. (B) ChIP-qPCR primers.
