# Supplementary figures and images for: Temporal requirements for ISL1 in sympathetic neuron proliferation, differentiation, and diversification
Source: Cell Death Dis. 2018 Feb 14;9(2):247. doi: 10.1038/s41419-018-0283-9 (PMC5833373; doi:10.1038/s41419-018-0283-9)

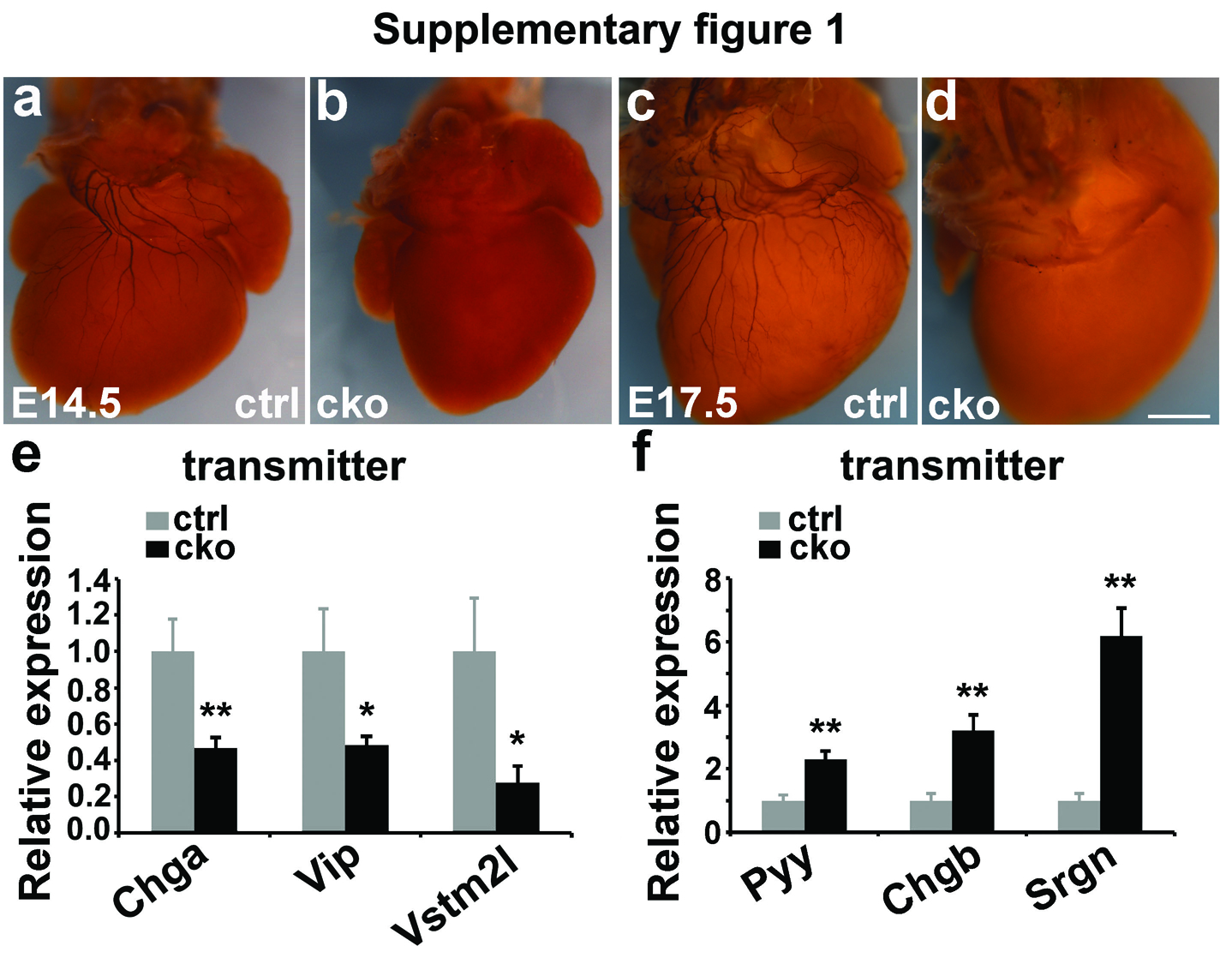

Supplement: Supplementary file 2 — Supplementary figure 1 [file 41419_2018_283_MOESM2_ESM.tif]

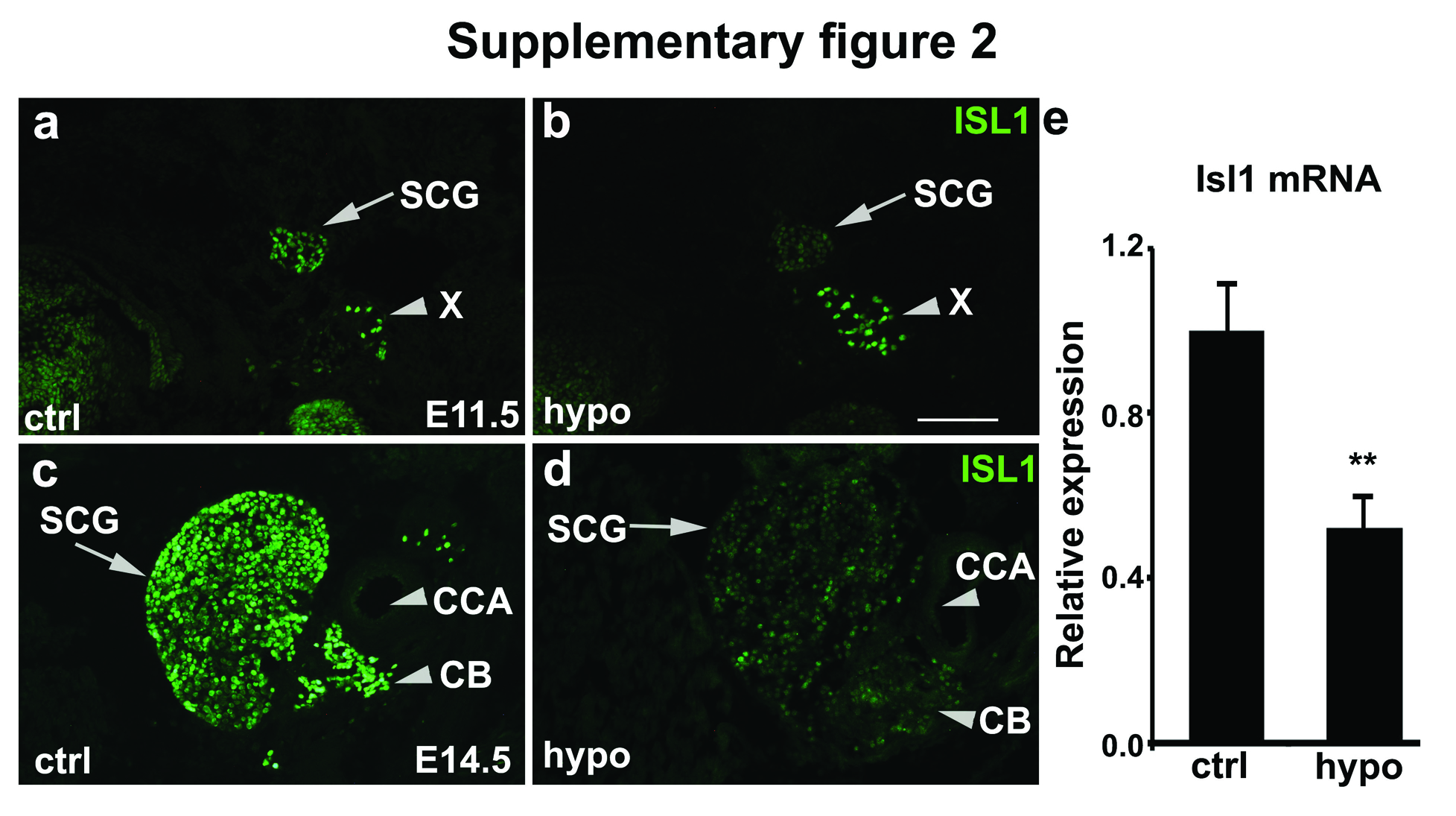

Supplement: Supplementary file 3 — Supplementary figure 2 [file 41419_2018_283_MOESM3_ESM.tif]

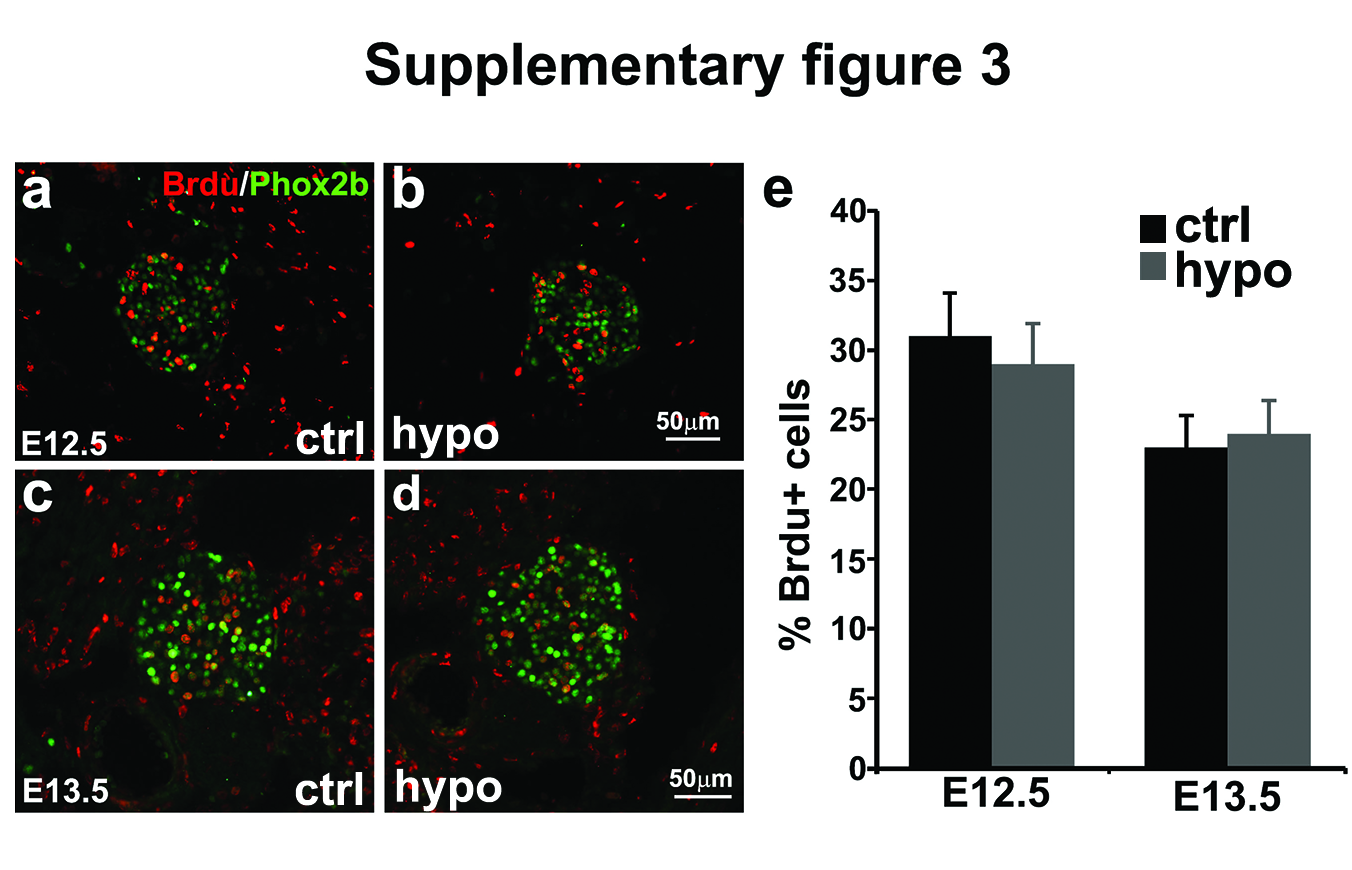

Supplement: Supplementary file 4 — Supplementary figure 3 [file 41419_2018_283_MOESM4_ESM.tif]

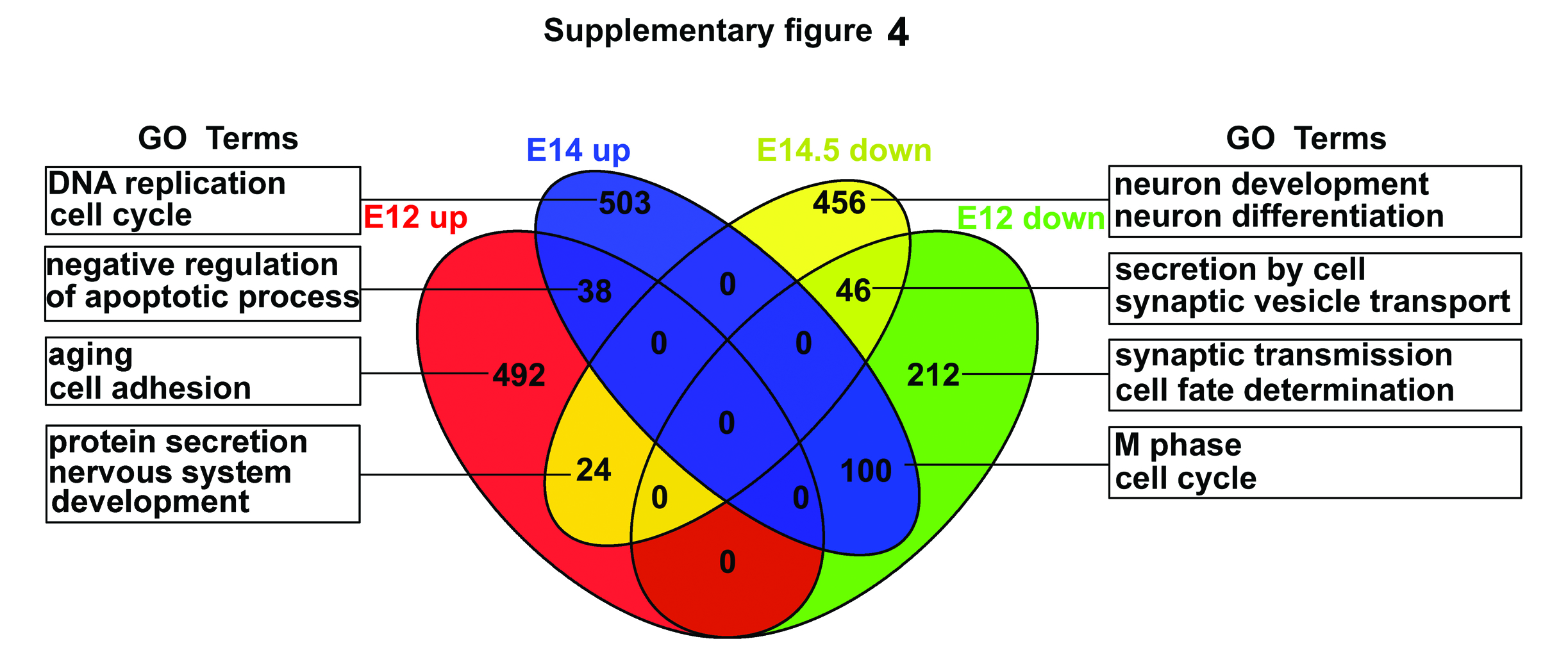

Supplement: Supplementary file 5 — Supplementary figure 4 [file 41419_2018_283_MOESM5_ESM.tif]

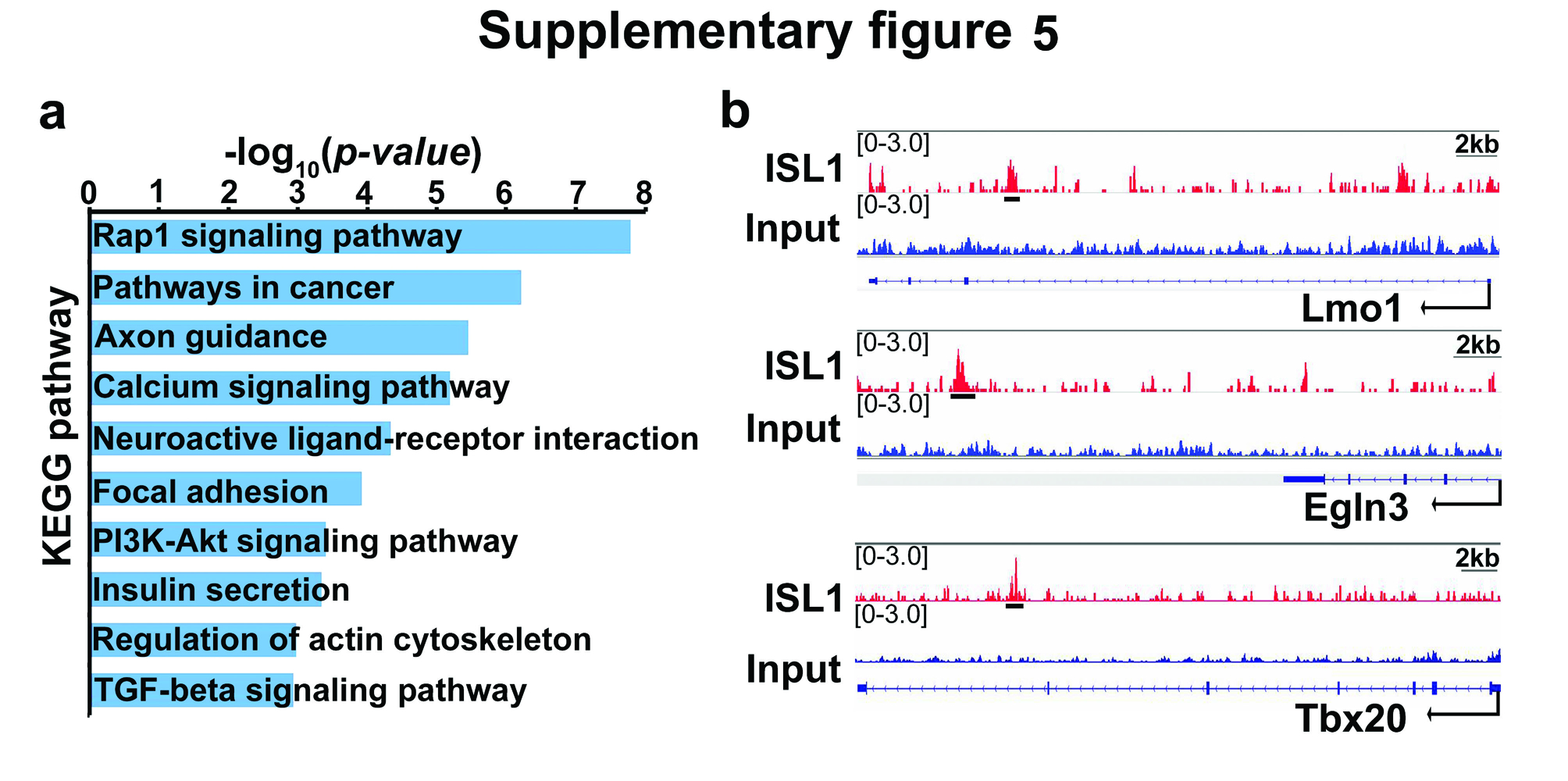

Supplement: Supplementary file 6 — Supplementary figure 5 [file 41419_2018_283_MOESM6_ESM.tif]

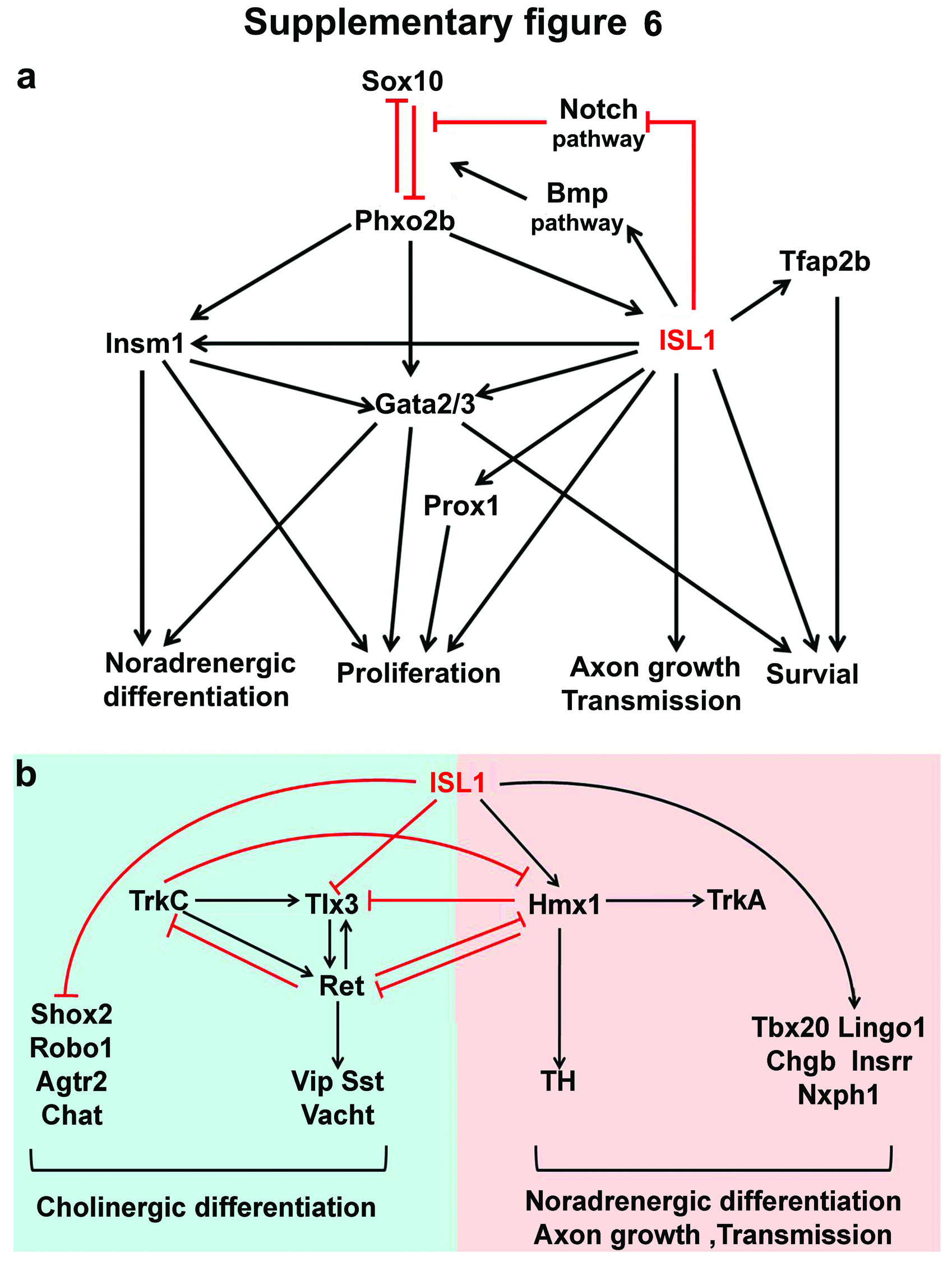

Supplement: Supplementary file 7 — Supplementary figure 6 [file 41419_2018_283_MOESM7_ESM.tif]
