# Supplementary material for: Temporal requirements for ISL1 in sympathetic neuron proliferation, differentiation, and diversification
Source: Cell Death Dis. 2018 Feb 14;9(2):247. doi: 10.1038/s41419-018-0283-9 (PMC5833373; doi:10.1038/s41419-018-0283-9)
Supplement: Supplementary file 12 — Supplementary Table 5 [file 41419_2018_283_MOESM12_ESM.pdf]

Table 5 Homer Known Motif Enrichment Results(ISL1 ChIP-seq peak)

Rank	Motif	Name	P-value	logP-value	q-value (Benjiamini)	# Target Sequences with Motif	%of Targets Sequences with Motif	Background Sequences with Motif	% of Background Sequences with Motif
1	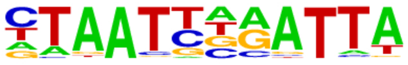	Phox2a(Homeobox)/Neuron-Phox2a-ChIP-Seq(GSE31456)/Homer	1e-60	-1.386e+02	0.0000	450.0	17.78%	3636.6	7.72%
2	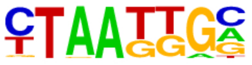	Isl1(Homeobox)/Neuron-Isl1-ChIP-Seq(GSE31456)/Homer	1e-48	-1.112e+02	0.0000	928.0	36.67%	11126.5	23.62%
3	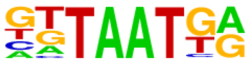	Nkx6.1(Homeobox)/Islet-Nkx6.1-ChIP-Seq(GSE40975)/Homer	1e-33	-7.805e+01	0.0000	1340.0	52.94%	19249.0	40.87%
4	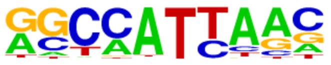	Nanog(Homeobox)/mES-Nanog-ChIP-Seq(GSE11724)/Homer	1e-24	-5.749e+01	0.0000	1396.0	55.16%	21105.0	44.81%
5	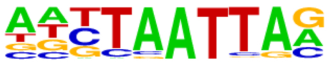	Lhx3(Homeobox)/Neuron-Lhx3-ChIP-Seq(GSE31456)/Homer	1e-19	-4.561e+01	0.0000	880.0	34.77%	12452.7	26.44%
6	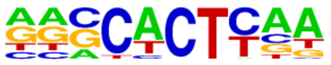	Nkx2.5(Homeobox)/HL1-Nkx2.5.biotin-ChIP-Seq(GSE21529)/Homer	1e-16	-3.889e+01	0.0000	612.0	24.18%	8237.7	17.49%
7	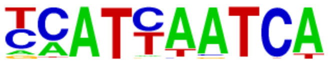	Pdx1(Homeobox)/Islet-Pdx1-ChIP-Seq(SRA008281)/Homer	1e-14	-3.352e+01	0.0000	498.0	19.68%	6594.3	14.00%
8	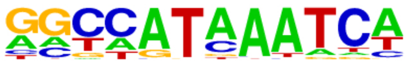	HOXA9(Homeobox)/HSC-Hoxa9-ChIP-Seq(GSE33509)/Homer	1e-13	-3.207e+01	0.0000	356.0	14.07%	4401.6	9.34%
9	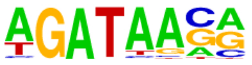	GATA3(Zf)/iTreg-Gata3-ChIP-Seq(GSE20898)/Homer	1e-13	-3.201e+01	0.0000	658.0	26.00%	9296.4	19.74%
10	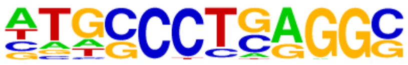	AP-2alpha(AP2)/Hela-AP2alpha-ChIP-Seq(GSE31477)/Homer	1e-13	-3.093e+01	0.0000	120.0	4.74%	1043.9	2.22%
11	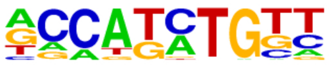	Olig2(bHLH)/Neuron-Olig2-ChIP-Seq(GSE30882)/Homer	1e-12	-2.927e+01	0.0000	515.0	20.35%	7045.3	14.96%
12	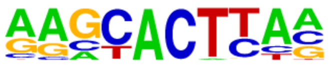	Nkx3.1(Homeobox)/LNCaP-Nkx3.1-ChIP-Seq(GSE28264)/Homer	1e-11	-2.591e+01	0.0000	673.0	26.59%	9853.6	20.92%
13	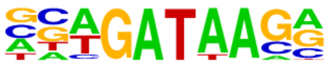	Gata4(Zf)/Heart-Gata4-ChIP-Seq(GSE35151)/Homer	1e-11	-2.549e+01	0.0000	419.0	16.55%	5643.2	11.98%
14	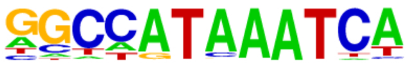	Hoxc9(Homeobox)/Ainv15-Hoxc9-ChIP-Seq(GSE21812)/Homer	1e-10	-2.413e+01	0.0000	282.0	11.14%	3526.8	7.49%
15	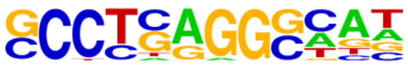	AP-2gamma(AP2)/MCF7-TFAP2C-ChIP-Seq(GSE21234)/Homer	1e-10	-2.361e+01	0.0000	141.0	5.57%	1458.7	3.10%
16	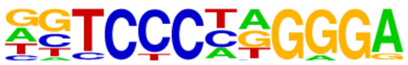	EBF(EBF)/proBcell-EBF-ChIP-Seq(GSE21978)/Homer	1e-10	-2.335e+01	0.0000	50.0	1.98%	319.4	0.68%
17	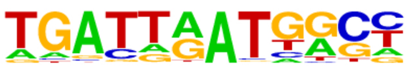	Hoxb4(Homeobox)/ES-Hoxb4-ChIP-Seq(GSE34014)/Homer	1e-9	-2.152e+01	0.0000	105.0	4.15%	1013.0	2.15%
18	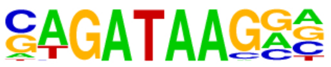	Gata1(Zf)/K562-GATA1-ChIP-Seq(GSE18829)/Homer	1e-9	-2.095e+01	0.0000	238.0	9.40%	2958.2	6.28%
19	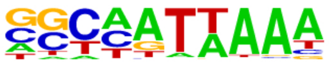	Unknown(Homeobox)/Limb-p300-ChIP-Seq/Homer	1e-8	-1.968e+01	0.0000	321.0	12.68%	4312.5	9.16%
20	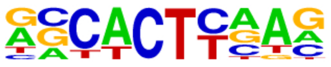	Nkx2.1(Homeobox)/LungAC-Nkx2.1-ChIP-Seq(GSE43252)/Homer	1e-8	-1.968e+01	0.0000	700.0	27.66%	10681.6	22.68%
21	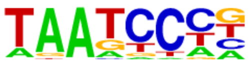	Pitx1(Homeobox)/Chicken-Pitx1-ChIP-Seq(GSE38910)/Homer	1e-8	-1.870e+01	0.0000	1338.0	52.86%	22242.0	47.22%
22	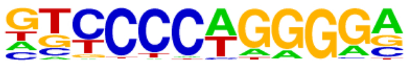	EBF1(EBF)/Near-E2A-ChIP-Seq(GSE21512)/Homer	1e-8	-1.850e+01	0.0000	145.0	5.73%	1640.2	3.48%
23	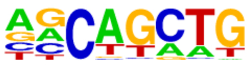	SCL(bHLH)/HPC7-Scl-ChIP-Seq(GSE13511)/Homer	1e-7	-1.810e+01	0.0000	915.0	36.15%	14581.0	30.96%
